# DNA methylation‐based telomere length is associated with HIV infection, physical frailty, cancer, and all‐cause mortality

**DOI:** 10.1111/acel.14174

**Published:** 2024-04-17

**Authors:** Xiaoyu Liang, Bradley E. Aouizerat, Kaku So‐Armah, Mardge H. Cohen, Vincent C. Marconi, Ke Xu, Amy C. Justice

**Affiliations:** ^1^ Department of Epidemiology and Biostatistics Michigan State University East Lansing Michigan USA; ^2^ Translational Research Center, College of Dentistry New York University New York New York USA; ^3^ Department of Oral and Maxillofacial Surgery, College of Dentistry New York University New York New York USA; ^4^ Boston University School of Medicine Boston Massachusetts USA; ^5^ Department of Medicine Stroger Hospital of Cook County Chicago Illinois USA; ^6^ Emory University School of Medicine and Rollins School of Public Health The Atlanta Veterans Affairs Medical Center Atlanta Georgia USA; ^7^ Department of Psychiatry Yale School of Medicine New Haven Connecticut USA; ^8^ VA Connecticut Healthcare System West Haven Connecticut USA; ^9^ Department of Internal Medicine Yale School of Medicine New Haven Connecticut USA; ^10^ Yale School of Public Health New Haven Connecticut USA

**Keywords:** all‐cause mortality, cancer, DNA methylation‐based telomere length, people with HIV, physiologic frailty

## Abstract

Telomere length (TL) is an important indicator of cellular aging. Shorter TL is associated with several age‐related diseases including coronary heart disease, heart failure, diabetes, osteoporosis, and cancer. Recently, a DNA methylation‐based TL (DNAmTL) estimator has been developed as an alternative method for directly measuring TL. In this study, we examined the association of DNAmTL with cancer prevalence and mortality risk among people with and without HIV in the Veterans Aging Cohort Study Biomarker Cohort (VACS, *N* = 1917) and Women's Interagency HIV Study Cohort (WIHS, *N* = 481). We profiled DNAm in whole blood (VACS) or in peripheral blood mononuclear cells (WIHS) using an array‐based method. Cancer prevalence was estimated from electronic medical records and cancer registry data. The VACS Index was used as a measure of physiologic frailty. Models were adjusted for self‐reported race and ethnicity, batch, smoking status, alcohol consumption, and five cell types (CD4, CD8, NK, B cell, and monocyte). We found that people with HIV had shorter average DNAmTL than those without HIV infection [beta = −0.25, 95% confidence interval (−0.32, −0.18), *p* = 1.48E‐12]. Greater value of VACS Index [beta = −0.002 (−0.003, −0.001), *p* = 2.82E‐05] and higher cancer prevalence [beta = −0.07 (−0.10, −0.03), *p* = 1.37E‐04 without adjusting age] were associated with shortened DNAmTL. In addition, one kilobase decrease in DNAmTL was associated with a 40% increase in mortality risk [hazard ratio: 0.60 (0.44, 0.82), *p* = 1.42E‐03]. In summary, HIV infection, physiologic frailty, and cancer are associated with shortening DNAmTL, contributing to an increased risk of all‐cause mortality.

## Graphical abstract

Telomere length (TL) serves as a key indicator of cellular aging, with shorter TL linked to various age‐related diseases. A DNA methylation‐based TL (DNAmTL) estimator has been developed as an alternative method for direct TL measurement. In this study, we observed that people living with HIV exhibit shorter DNAmTL. Furthermore, a reduction of DNAmTL is linked to greater physical frailty, higher prevalence of cancer, and a heightened risk of all‐cause mortality in our studied population.

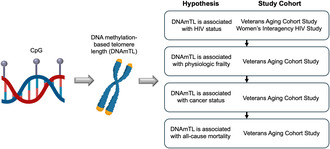

**Abbreviations**
450K: Illumina HumanMethylation450 BeadChip
AA: African American
ART: antiretroviral therapy
AUDIT‐C: first three questions of the alcohol use disorders identification test
BMI: body mass index
bp: base pair
CI: confidence interval
CpG: cytosine‐phosphate‐guanine dinucleotide
DNAm: DNA methylation
DNAmGrim: Lu's GrimAge clock
DNAmHannum: Hannum clock
DNAmHorvath: Horvath clock
DNAmMono: monocyte clock
DNAmPheno: Levine clock
DNAmTL: DNA methylation‐based telomere length
DNAmTLadjAge: age‐adjusted estimate of DNAmTL
DunedinPoAm: Dunedin pace of aging methylation clock
EAA: epigenetic age acceleration
EEAA: extrinsic epigenetic age acceleration
EPIC: Illumina HumanMethylation EPIC BeadChip
flow FISH: flow cytometry‐fluorescence in situ hybridization
HCV: hepatitis C infection
HR: hazard ratio
IEAA: intrinsic epigenetic age acceleration
lnPEth: natural logarithm of phosphatidylethanol
log10VL: log10 of viral load
MD: mean difference
NDRNKWK: number of drinks per week
PBMC: peripheral blood mononuclear cell
PCA: principal component analysis
PWH: people with HIV infection
PWoH: people without HIV infection
QC: quality control
qPCR: quantitative PCR
SNP: single nucleotide polymorphism
TL: telomere length
TRF: terminal restriction fragment
TRF‐TL: TRF‐based estimate of TL
VACS Index: VACS Index 2.0
VACS: veteran aging cohort study
VACS1: VACS cohort 1
VACS2: VACS cohort 2
VAMC: veterans affairs medical center
WIHS: women's interagency HIV study

## 1 INTRODUCTION

People with HIV infection (PWH) are more susceptible to age‐related health problems compared to the general population. Extensive research has definitively demonstrated a higher prevalence of comorbidity and multimorbidity among PWH, which can be attributed to factors including the biological effects of HIV infection, the side effects of antiretroviral therapy (ART), and premature aging (Calcagno et al., 2015; Maciel et al., 2018; Marcus et al., 2020; Pathai et al., 2013; Roomaney et al., 2022). For instance, older PWH are at a higher risk of experiencing cardiovascular disease, dementia, diabetes, osteoporosis, frailty, and specific types of cancers (Biver, 2022; Carbone et al., 2022; Falutz et al., 2021; McArthur, 2004; Noubissi et al., 2018; Roomaney et al., 2022; So‐Armah et al., 2020). Relatedly, the prevalence of cancer tends to increase with age, indicating that adult forms of cancer can be regarded as an age‐associated condition (White et al., 2014).

Telomeres, the nucleoprotein structures that cap the ends of linear chromosomes, play a crucial role in maintaining chromosome integrity by preventing fraying or tangling of the chromosome ends (Capper et al., 2007; Fasching, 2018). Telomeres naturally shorten with each cell division. As telomeres become shorter, it can lead to genomic instability, cellular senescence, and impaired cellular function (Rossiello et al., 2022). Eventually, when telomeres become too short, cells no longer divide successfully, leading to oncogenesis or cell death (Mai & Garini, 2006). Telomere shortening is a well‐known hallmark of both cellular senescence and physiological aging and has been associated with detrimental effects on health (Vaiserman & Krasnienkov, 2020). Numerous age‐related diseases and premature aging syndromes exhibit an accelerated rate of telomere shortening, implicating telomere shortening is a component of the aging process (Blasco, 2007). For example, telomere length (TL) is associated with the incidence, progression, and disease‐specific mortality of age‐related diseases, including cardiovascular disease (i.e., myocardial infarction) (Pusceddu et al., 2018), type 2 diabetes (Salpea et al., 2010), Alzheimer's disease (Fani et al., 2020), and cancer (Maciejowski & de Lange, 2017, 2019). TL is widely accepted as a robust biomarker of aging and age‐related pathological conditions (Fasching, 2018). The value of TL as an aging biomarker stems from its correlation with chronological age across the lifespan, its predictive utility for disease occurrence and mortality, as well as its significant responsiveness to both detrimental and beneficial exposures (Hastings et al., 2017).

Studies have shown statistically significant associations between TL and both cancer incidence and mortality (Willeit et al., 2010). For instance, previous research has demonstrated a link between short TL and bladder cancer (Broberg et al., 2005; McGrath et al., 2007; Wu et al., 2003), renal cell carcinoma (Shao et al., 2007; Wu et al., 2003), non‐Hodgkin lymphoma (Lan et al., 2009), lung cancer (Jang et al., 2008), head and neck tumors (Wu et al., 2003), colorectal cancer, breast cancer (Lee et al., 2010; Shen et al., 2009; Zee et al., 2009), esophageal, gastric, ovarian cancers, and overall incident cancer (Zhang et al., 2015). Shorter TL is associated with increased all‐cause mortality risk in the general population (Wang et al., 2018) and can serve as an independent prognostic predictor of survival in patients, including those with bladder cancer (Allaire et al., 2023; Russo et al., 2014). Furthermore, different biological pathways, including hormone metabolism, tobacco carcinogen metabolism, and DNA damage repair mechanisms, may interact with telomere length, leading to differential impacts on cancer susceptibility (Ma et al., 2011). For example, some studies demonstrated that the influence of shortened telomeres on breast cancer risk may be greater in specific subgroups, such as premenopausal women and those with compromised antioxidative capacity but may not be significant in the overall study population or among postmenopausal women (De Vivo et al., 2009; Shen et al., 2009; Zheng et al., 2010). Together, the evidence demonstrates the inverse relationship between TL and the variety of cancer diagnoses.

The proliferation of studies examining telomeres as a biomarker in physiological, psychological, and biobehavioral research has driven the development of many methods and technologies to assess TL (Kimura et al., 2010; Lai et al., 2018; Mender & Shay, 2015; Montpetit et al., 2014). However, the reliable measurement of TL remains challenging for technical reasons, including but not limited to variation in methods employed for DNA extraction and the high level of technical expertise required, regardless of the specific method utilized (Lu, Seeboth, et al., 2019; Nussey et al., 2014).

DNA methylation (DNAm) of cytosine‐phosphate‐guanine dinucleotides (CpGs), an epigenetic mechanism regulating gene expression, is another DNA‐based biomarker that exhibits age‐related alterations. These alterations in DNAm have been suggested to be useful biomarkers for diagnosis, prognosis, and treatment response (Horvath & Raj, 2018; Muller & Gyorffy, 2022). Through machine learning techniques, epigenetic age estimators have been developed to accurately estimate biological age (referred to as DNAm age) in different tissues throughout an individual's lifespan (Hannum et al., 2013; Horvath, 2013; Horvath & Raj, 2018; Levine et al., 2018; Liang et al., 2022; Lu, Quach, et al., 2019). Recent research suggests that epigenetic age holds the most promise as a molecular estimator of biological age (Horvath & Raj, 2018; Jylhava et al., 2017). Given the evidence of TL‐related DNAm changes (Hu et al., 2018), Lu et al. developed a novel DNAm‐based TL estimator (DNAmTL) based on methylation profiles of 140 CpGs (Lu, Seeboth, et al., 2019). They regressed TL that was measured using the terminal restriction fragment (TRF) assay (Mender & Shay, 2015) on DNAm isolated from whole blood using an elastic net regression model, resulting in identification of a set of 140 CpGs whose methylation levels best predicted TRF‐based estimate of TL (TRF‐TL). DNAmTL shares the same units (kilobases) as the TRF‐TL. It has been validated as a surrogate of TL. It is positively correlated with TRF‐TL (*r* = 0.38 ~ 0.5 in the validation data sets) (Lu, Seeboth, et al., 2019), quantitative PCR (qPCR) TL (*r* = 0.41), and flow cytometry‐fluorescence in situ hybridization (flow FISH) TL (*r* = 0.56) (Pearce et al., 2021), suggesting that DNAmTL is consistent with other methods and is a reliable estimator of TL. Studies have also shown that DNAmTL was negatively correlated with age across different tissues and cell types and outperformed the TRF‐TL method in predicting all‐cause mortality [DNAmTL: hazard ratio (HR) = 0.31 and *p* = 6.7E‐09, TRF‐TL: HR = 0.81 and *p* = 4.70E‐03], time‐to‐coronary heart failure (DNAmTL: HR = 0.55 and *p* = 9.50E‐03, TRF‐TL: not significant), and its associations with smoking history (DNAmTL: beta = 0.08 and *p* = 2.09E‐04, TRF‐TL: beta = 0.13 and *p* = 3.50E‐02), and other age‐related conditions (Lu, Seeboth, et al., 2019). Therefore, DNAmTL serves as a reasonably performing indirect estimator of TL when direct TL methods are challenging or unavailable.

In addition to DNAmTL, other epigenetic clocks have been established, including the Monocyte clock (DNAmMono, based on 186 CpGs from monocyte methylomes) (Liang et al., 2022), the Horvath clock (DNAmHorvath, based on 353 CpGs that capture estimated multi‐tissue biological age) (Horvath, 2013), the Hannum clock (DNAmHannum, based on 71 CpGs in leukocytes) (Hannum et al., 2013), the Levine clock (DNAmPheno, based on 513 CpGs to predict lifespan) (Levine et al., 2018), the Lu's GrimAge clock (DNAmGrim, a linear combination of chronological age, sex, and 1030 CpG sites modeled as surrogate biomarkers for seven plasma proteins and smoking pack‐years, predicting age at death) (Lu, Quach, et al., 2019), the Dunedin Pace of Aging methylation clock (DunedinPoAm, based on 46 CpGs to predict the Pace of Aging) (Belsky et al., 2020), and two widely used measures of age acceleration, intrinsic epigenetic age acceleration (IEAA) and extrinsic epigenetic age acceleration (EEAA) (Chen et al., 2016; Okazaki et al., 2020; Smith et al., 2019). IEAA, which is based on Horvath's clock, captures cellular age acceleration independently of blood cell counts, indicates cell‐intrinsic aging. On the other hand, EEAA, which is based on Hannum's clock, is associated with age‐dependent changes in blood cell counts and indicates immune system aging (Chen et al., 2016; Okazaki et al., 2020; Smith et al., 2019).

While DNAmTL demonstrates potential as a robust estimator of TL, its use in studies of the relationships between TL and chronic health conditions or aging‐related diseases remains relatively novel, especially in the scenario where direct TL measurements are unavailable. In this study, we utilized the DNAmTL as a new approach to investigate the associations between TL with HIV infection, physiologic frailty, cancer, and all‐cause mortality. The Veteran Aging Cohort Study (VACS) served as the primary cohort for the analyses and the Women's Interagency HIV Study (WIHS) served as a secondary cohort to replicate the observed association between DNAmTL and HIV infection. We used multivariable linear regression to estimate the association of DNAmTL with cancer and physiologic frailty. We utilized Cox proportional hazards models to assess an association of DNAmTL with the risk of all‐cause mortality. The hypotheses and corresponding cohorts of our study are provided in Figure S1. Employing a measure of TL that is less susceptible to the technical challenges of assays that directly measure TL has the potential to advance our understanding of the intricate connections between TL, HIV infection, physiologic frailty, cancer, and the aging process.

## 2 METHODS

### 2.1 Study cohorts and phenotype assessments

#### 2.1.1 Veterans aging cohort study (VACS) (*N* = 1917)

The VACS (Table 1) is an ongoing longitudinal, prospective, and multisite observational cohort study across multiple sites, aiming to examine various health outcomes, disease progression, and treatment responses in both PWH and people without HIV infection (PWoH) receiving care at Veterans Affairs Medical Center infectious disease and general medical clinics (Justice et al., 2006). Written consent was obtained from all participants. Within a subset of the cohort, biospecimens comprising DNA samples obtained from whole blood were collected.

**TABLE 1:** Demographic and clinical characteristics of VACS.

Phenotype	VACS1						VACS2			
	PWH	PWoH	*p*‐Value	Cancer+	Cancer−	*p*‐Value	PWoH	Cancer+	Cancer−	*p*‐Value
	(*N* = 1147)	(*N* = 104)		(*N* = 286)	(*N* = 888)		(*N* = 666)	(*N* = 135)	(*N* = 479)	
Age	51.08 ± 7.69	53.35 ± 8.88	**1.30E‐02**	52.92 ± 7.31	50.60 ± 7.86	**5.66E‐06**	53.74 ± 9.18	56.36 ± 7.84	52.88 ± 9.53	**2.10E‐05**
BMI	25.43 ± 4.40	29.69 ± 6.03	**1.61E‐10**	25.36 ± 4.53	25.94 ± 4.79	6.27E‐02	29.24 ± 5.73	28.73 ± 5.61	29.36 ± 5.73	2.50E‐01
Sex (male)	100%	100%	NA	100%	100%	NA	100%	100%	100%	NA
Self‐reported race (AA)^a^	83.52%	61.54%	**2.84E‐08**	84.62%	80.86%	1.52E‐01	67.72%	71.11%	66.18%	2.81E‐01
Smoker	58.96%	51.46%	1.39E‐01	58.80%	57.78%	7.62E‐01	49.77%	53.38%	48.06%	2.79E‐01
lnPEth^b^	1.94 ± 1.80	1.72 ± 1.71	2.69E‐01	1.91 ± 1.87	1.92 ± 1.76	9.41E‐01	2.04 ± 1.99	2.42 ± 2.11	1.93 ± 1.94	**2.38E‐02**
Alcohol^c^	3.31 ± 2.63	3.81 ± 2.83	1.78E‐01	3.32 ± 2.65	3.35 ± 2.64	8.90E‐01	3.68 ± 2.85	4.18 ± 2.88	3.61 ± 2.85	9.95E‐02
VACS Index	31.03 ± 19.95	NaN ± NA	NA	32.84 ± 19.76	30.46 ± 20.00	9.19E‐02	NaN ± NA	NaN ± NA	NaN ± NA	NA
log10VL^d^	2.67 ± 1.22	NaN ± NA	NA	2.55 ± 1.16	2.71 ± 1.24	6.11E‐02	2.01 ± 0.40	NaN ± NA	2.01 ± 0.40	NA
ART adherence^e^	77.62%	0%	**1.30E‐62**	75%	69.53%	7.97E‐02	0%	0%	0%	NA
Cannabis use^f^	78.77%	63%	**2.98E‐04**	79%	77.03%	4.91E‐01	64.97%	66.42%	65.66%	8.70E‐01
Cocaine use^f^	70.35%	52.04%	**1.72E‐04**	70.32%	67.93%	4.53E‐01	51.94%	50.75%	53.04%	6.39E‐01
Opioid use^f^	45.24%	33.67%	**2.71E‐02**	45.74%	43.06%	4.29E‐01	35.50%	34.07%	36.52%	6.02E‐01
Stimulant use^f^	39.96%	30.61%	6.90E‐02	40.57%	37.89%	4.23E‐01	32.19%	34.59%	32.31%	6.23E‐01
CD4T	0.06 ± 0.05	0.13 ± 0.06	**6.78E‐20**	0.07 ± 0.06	0.07 ± 0.06	5.49E‐01	0.16 ± 0.06	0.17 ± 0.05	0.16 ± 0.06	2.72E‐01
CD8T	0.17 ± 0.08	0.07 ± 0.05	**1.13E‐42**	0.17 ± 0.08	0.16 ± 0.08	2.80E‐01	0.04 ± 0.04	0.04 ± 0.04	0.04 ± 0.04	6.25E‐01
Granulocyte	0.51 ± 0.12	0.61 ± 0.13	**1.07E‐10**	0.51 ± 0.13	0.52 ± 0.13	2.72E‐01	0.58 ± 0.11	0.56 ± 0.10	0.58 ± 0.11	7.13E‐02
NK	0.08 ± 0.06	0.07 ± 0.05	**1.07E‐02**	0.08 ± 0.06	0.08 ± 0.06	6.98E‐02	0.07 ± 0.05	0.07 ± 0.05	0.07 ± 0.05	9.22E‐01
B cell	0.10 ± 0.05	0.07 ± 0.04	**2.04E‐08**	0.09 ± 0.05	0.10 ± 0.05	4.13E‐01	0.07 ± 0.04	0.08 ± 0.04	0.07 ± 0.04	1.39E‐01
Monocyte	0.11 ± 0.04	0.10 ± 0.03	**2.89E‐04**	0.11 ± 0.04	0.11 ± 0.04	9.12E‐01	0.09 ± 0.03	0.09 ± 0.04	0.09 ± 0.03	9.34E‐02

*Note*: Welch's two‐sample *t*‐test was used to compare means between two groups; chi‐square test was used to compare percentages between two groups. Significant phenotypes are shown in bold.

^a^ AA, African American.

^b^ lnPEth: natural logarithm of phosphatidylethanol, an objective measure of alcohol consumption.

^c^ Alcohol: AUDIT‐C (first three questions of the Alcohol Use Disorders Identification Test) in VACS and NDRNKWK (number of drinks per week) in WIHS.

^d^ log10VL: log10 of viral load.

^e^ ART adherence: adherence to antiretroviral therapy.

^f^ The cannabis, cocaine, stimulant, and opioid use were defined as a case (>0) and a control (=0). In WIHS, instead of individual measures for opioid or cocaine use, the phenotype is based on the cumulative use of opioids or cocaine.

The VACS biomarker cohort included two subsets of the samples, VACS cohort 1 (VACS1) (*N* = 1251) and VACS cohort 2 (VACS2) (*N* = 666). VACS1 included both PWH and PWoH while VACS2 included only PWoH. Both cohorts served as clinic‐based samples to analyze DNAm profiles. In VACS1, 286 participants had a cancer diagnosis (cancer+) while 888 participants did not (cancer‐). VACS2 included 666 samples obtained from PWoH, of which 135 participants had cancer, while 479 participants did not. Fifty‐two participants had no information. Due to the limited sample size, we were unable to examine the relationship between DNAmTL and specific cancer diagnoses. In our analysis, the “cancer+” designation indicates participants who have been diagnosed with cancer of any type regardless of remission/treatment. The cancer diagnosis was based on ICD‐10 criteria, and the data were extracted from electronic medical records in the Veterans Affairs of Healthcare System in the United States.

HIV physiological frailty was measured by the VACS Index which was developed from a cohort of US Veterans with HIV and has been validated in several European and North American cohorts (Justice et al., 2012, 2013; Tate et al., 2013). Similar to the Clinical Frailty Scale developed by Rockwood et al. (Rockwood Frailty Index) (Rockwood et al., 2005; Rockwood & Mitnitski, 2007), the VACS Index considers frailty as an accumulation of deficits, including general indicators of organ system injury such as hemoglobin and platelet counts (Moore et al., 2023). However, it also includes indicators specific to HIV, such as CD4 T‐cell count, and plasma HIV‐1 RNA. While the Rockwood Frailty Index is designed to access a generalized, accelerated aging process, the VACS Index is primarily aimed at predicting hospitalization and all‐cause mortality specifically in PWH (Moore et al., 2023). Nonetheless, the VACS Index contains a number of components that fit the conceptual framework of frailty. It is a weighted score calculated by summing preassigned points for age, eight routinely monitored indicators of HIV disease, including CD4 T‐cell count and plasma HIV‐1 RNA, and other general indicators of organ system injury, including hemoglobin, aspartate and alanine transaminase, platelet count, creatinine, and viral hepatitis C infection (HCV). Individuals with a high VACS Index tend to have poorer HIV‐related outcomes, and higher VACS Index scores are linked to an increased risk of mortality. The VACS Index provides a comprehensive overview of an individual's health, making it a valuable tool for guiding both clinical decisions and research endeavors in the context of HIV and aging. In addition to the VACS Index, we also included the duration of infection, HIV‐1 viral load, and ART adherence information for PWH.

The date and cause of death for the VACS participants were obtained from the Veteran Health Administration vital status file. The file integrates information from multiple sources, including the Social Security Administration death master file, the Beneficiary Identification and Records Locator Subsystem, and the Veteran Health Administration Medical Statistical Analysis Systems inpatient datasets.

#### 2.1.2 Women's interagency HIV study (*N* = 481)

The WIHS (Table S1) is the world's largest and longest running prospective cohort study dedicated to the study of the natural and treated history of women living with HIV and includes a risk‐set matched comparison group of women without HIV infection, offering valuable insights into their health outcomes (Adimora et al., 2018; Bacon et al., 2005). The WIHS cohort, consisting of individuals from both clinic and community settings, was utilized to investigate the impact of HIV infection on DNAmTL. The study included both PWH (*N* = 272) and PWoH (*N* = 209). The WIHS cohort served as a replication sample for the association of DNAmTL with HIV infection. Cancer diagnosis, VACS Index, and mortality in the WIHS cohort were unavailable for the current study.

### 2.2 DNAm and data quality control (QC)

Epigenome‐wide DNAm levels from VACS whole blood samples were assessed using two platforms: Illumina HumanMethylation450 BeadChip (450K) (VACS1) and Illumina HumanMethylation EPIC BeadChip (EPIC) (VACS2). For WIHS samples, DNAm levels were measured from peripheral blood mononuclear cells (PBMCs) using the EPIC. All samples were processed at the Yale Center for Genomic Analysis (Zhang et al., 2017), following the same QC criteria as our previous studies (Xu et al., 2018; Zhang et al., 2017, 2016). We retrieved raw methylation data and performed downstream analysis using the minfi R package (version 1.18.1). Normalization was separately performed for the 450K and EPIC methylation datasets. For the 450K dataset, quantile normalization was performed on six separated intensity values following the method outlined by Lehne et al. (Lehne et al., 2015). For the EPIC dataset, the ssNoob method was applied (Fortin et al., 2017). Detection p‐value thresholds of <1e−12 and <1e‐8 were set to enhance the quantification of methylation intensities for the 450K and EPIC datasets, respectively, using probes on the Y chromosome. CpG sites on the sex chromosomes (X: *N* = 11,232 CpGs, Y: *N* = 416 CpGs) as well as those with annotated a single nucleotide polymorphism (SNP) within 10 bp (*N* = 47,790) were excluded from the analysis. Additionally, previously identified cross‐reactive probes were excluded (Chen et al., 2013). This yielded a total of 437,722 CpG sites on the 450K array and 846,604 CpG sites on the EPIC array remained for the analysis. Three samples with a call rate <98% were also excluded. We compared the predicted sex with the self‐reported sex, and mismatched samples were excluded (Heiss & Just, 2018).

### 2.3 Statistical analysis

#### 2.3.1 Consideration of confounding factors

Research suggests that individuals who self‐identify as African Americans (AAs) tend to have longer TL than Whites at birth but undergo a more rapid rate of telomere attrition throughout their lives (Brown et al., 2017; Rewak et al., 2014; Thomas et al., 2021). Higher body mass index (BMI) and obesity are associated with shortened TL (Gielen et al., 2018; Khosravaniardakani et al., 2022). Additionally, excessive alcohol consumption may adversely affect TL (Jung et al., 2022; Latifovic et al., 2016). Cigarette smoking and vigorous physical activity have an impact on TL (Astuti et al., 2017; Latifovic et al., 2016). A study involving a female cohort demonstrated that each pack‐year smoked corresponded to an additional five base pairs (bp) of telomere length lost (18%) compared to the overall cohort (Valdes et al., 2005). Therefore, in examining the relationships of DNAmTL and the phenotypes of interest, all statistical models accounted for potential confounding factors by adjusting for self‐reported race and ethnicity, BMI, assay sample batch, smoking status, alcohol consumption [natural logarithm of phosphatidylethanol (lnPEth) for VACS and number of drinks per week (NDRNKWK) for WIHS]. Six cell types (CD4+ T cells, CD8+ T cells, NK T cells, B cells, monocytes, and granulocytes) in the blood were estimated in each sample using the method described by Houseman et al. (Houseman et al., 2012). We found that DNAmTL and six other epigenetic clocks were significantly correlated with the six cell types regardless of HIV infection status, HIV physiologic frailty levels, and cancer status (Figure S2). Given the observed relationship between DNAmTL and cell type proportion, five cell type proportions (CD4+ T cells, CD8+ T cells, NK T cells, B cells, and monocytes) were considered as confounding factors and adjusted for in all subsequent statistical models.

#### 2.3.2 Calculation of epigenetic clocks

In addition to DNAmTL, we considered six well‐established epigenetic clocks and two measures of age acceleration in our study. The Monocyte clock (DNAmMono) is based on 186 CpGs from monocyte methylomes (Liang et al., 2022). The Horvath clock (DNAmHorvath) is based on 353 CpGs that capture estimated multi‐tissue biological age (Horvath, 2013). The Hannum clock (DNAmHannum) is based on 71 CpGs in leukocytes (Hannum et al., 2013). The Levine clock (DNAmPheno) is based on 513 CpGs to predict lifespan (Levine et al., 2018). The Lu's GrimAge clock (DNAmGrim) is a linear combination of chronological age, sex, and 1030 CpG sites modeled as surrogate biomarkers for seven plasma proteins and smoking pack‐years, predicting age at death (Lu, Quach, et al., 2019). The Dunedin Pace of Aging methylation clock (DunedinPoAm) selected 46 CpGs to predict the Pace of Aging in Dunedin Study (Belsky et al., 2020). The Pace of Aging is a combination of rates of change across 18 blood‐chemistry and organ‐system‐function biomarkers. Besides the epigenetic clocks, we incorporated two widely used measures of age acceleration. The intrinsic epigenetic age acceleration (IEAA) is the residual resulting from regressing DNAmHorvath on chronological age and blood cell count estimates (Chen et al., 2016). The extrinsic epigenetic age acceleration (EEAA) is the residual from regressing enhanced‐DNAmHannum onto chronological age. The enhanced‐DNAmHannum is a weighted average of DNAmHannum with three cell types that are known to change with age (Chen et al., 2016).

#### 2.3.3 Use of epigenetic age acceleration (EAA) in models of epigenetic age

For the various epigenetic clocks, including DNAmMono, DNAmHorvath, DNAmHannum, DNAmPheno, DNAmGrim, and DunedinPoAm, the EAA is defined as the residual obtained from regressing DNAm age on chronological age (Hannum et al., 2013; Horvath, 2013; Levine et al., 2018; Liang et al., 2022; Lu, Quach, et al., 2019). This measure helps determine whether individuals appear biologically younger or older than their chronological age. Specifically, a positive or negative value of EAA indicates that the DNAm age predicted from the chronological age is either accelerating (which is unfavorable) or decelerating (considered favorable), respectively.

#### 2.3.4 Calculation of age‐adjusted estimate of DNAmTL (DNAmTLadjAge)

The DNAmTLadjAge is defined as the residual of regressing DNAmTL on chronological age. Unlike the other epigenetic clocks, DNAmTL is negatively associated with chronological age. Therefore, if the resulting residual, DNAmTLadjAge, was positive or negative, it indicated that DNAmTL was either longer or shorter than that expected based on the chronological age, respectively (Shinko et al., 2022).

#### 2.3.5 Association analyses for outcomes of interest

Considering the negative association between DNAmTL and chronological age, and the acknowledged significance of chronological age as a crucial factor impacting DNAmTL, we decided to fit two models. Instead of utilizing DNAmTLadjAge, we performed a regression of DNAmTL on the phenotype of interest with and without age as a covariate in the model. This approach allowed us to directly assess how DNAmTL relates to the phenotype of interest while simultaneously accounting for age and other potential confounding factors. Employing these two models permitted us to evaluate the relationships between DNAmTL and the various predictors while acknowledging the critical role of chronological age in the analysis. Therefore, except for the lollipop plot and Kaplan–Meier curve using DNAmTLadjAge, all other analyses of DNAmTL include age as a covariate in the model. To correct for multiple testing, we additionally applied Bonferroni adjustment, incorporating the Bonferroni adjusted *p*‐value. This adjusted *p*‐value ($p_{\text{Bonf}}$) is calculated by multiplying the original *p*‐value by the number of tests conducted (nine methods) and by two models (adjusting for age and without adjusting for age).

To assess potential batch effects in our study, we applied principal component analysis (PCA) to the combined DNAm data of VACS1 and VACS2. The PCA scatter plot (Figure S3) visually revealed distinct clustering of samples from PWH and PWoH (Figure S3 left), indicating the presence of two different batches of VACS1 and VACS2, respectively. Thus, to reduce the batch effect, we performed the association of DNAmTL and HIV infection in the VACS1 only. However, the participants with and without cancer were evenly distributed in the two batches (Figure S3 right), suggesting little effect of batch on cancer diagnosis. Therefore, we analyzed the association of DNAmTL and cancer in all samples including VACS1 and VACS2. We then performed linear regression analyses to investigate the association between DNAmTL and the phenotypes of interest. Specifically, DNAmTL was treated as the dependent variable, while HIV, VACS Index, or cancer served as the independent variable, adjusting potential confounding factors. Following these analyses, we utilized *t*‐test to assess the significance of the regression coefficient derived from the linear regression models.

*Models of HIV infection in relation to DNAmTL* were fit using VACS1. As mentioned above, we tested an association between DNAmTL and HIV infection in WIHS to replicate the findings in the VACS1 cohort.

*Models of VACS Index in relation to DNAmTL* were fit using VACS1. The VACS Index is specifically designed for PWH. Therefore, our model was conducted to examine the association between DNAmTL and the VACS Index within the VACS1 cohort.

*Models of cancer in relation to DNAmTL* were fit in VACS: We examined the overall impact of DNAmTL and cancer status in all VACS samples.

*Models of mortality in relation to DNAmTL* were fit using VACS. We examined DNAmTL on all‐cause mortality using Cox regression analysis in all VACS samples. Before conducting the Cox regression analysis, we first tested the proportional hazard assumption to ensure its validity. Following the verification of the proportional hazard assumption, we proceeded with the Cox proportional hazards regression survival analysis to estimate the HR of DNAmTL and cancer status on all‐cause mortality, while controlling for relevant covariates. By adjusting for these covariates, we aimed to isolate the specific effect of DNAmTL and cancer on mortality, accounting for potential confounding factors.

## 3 RESULTS

### 3.1 Study cohorts and phenotype assessments

In VACS, 63.66% of PWH achieved viral suppression through ART while 90.44% of PWH in WIHS achieved viral suppression. Alcohol consumption was evaluated using two measures: the scores from the first three questions of the Alcohol Use Disorders Identification Test (AUDIT‐C) and the levels of PEth, which is a biomarker indicating alcohol use (Viel et al., 2012). It is worth noting that PEth levels have been found to have a positive correlation with AUDIT‐C scores (Liang et al., 2020; Piano et al., 2015). The average PEth level observed in the study was 41.7 ng/mL. Based on a previous study, hazardous alcohol drinking was defined as PEth level ≥20 ng/mL using the 16:0/18:1 test (Aboutara et al., 2023; Stewart et al., 2009). Within the WIHS cohort, the majority of participants reported light alcohol consumption, with an average 0.7 NDRNKWK (Adams et al., 1996). A summary of the participants' demographic and clinical characteristics of VACS and WIHS are presented in Table 1 and Table S1, respectively.

Figure S4 illustrates the distribution of DNAmTL within each subset of the samples separately. In the VACS1 cohort, the range of DNAmTL values spanned from 5.66 to 8.32, with a mean of 7.17. For the VACS2 cohort, DNAmTL exhibited a range between 6.07 and 7.88, and the mean value was calculated as 7.01. In the WIHS cohort, the DNAmTL range extended from 5.87 to 8.06, with a mean value of 7.09. Although there were significant differences in mean DNAmTL among the three subsets of the samples (ANOVA *F*‐test for comparing three group means: *p* = 1.271E‐21. *t*‐test for comparing each pair of group means: VACS1 vs. VACS2: *p* = 2.36E‐22; VACS1 vs. WIHS: *p* = 1.88E‐05; VACS2 vs. WIHS; *p* = 5.79E‐05), the distributions of DNAmTL among three sets of samples were similar.

### 3.2 A shortened DNAmTL associated with HIV infection and physiologic frailty

We examined the association between DNAmTL and HIV in both the VACS1 and WIHS cohorts (as presented in Table 2). In the VACS1 cohort, we found a significant relationship between DNAmTL and HIV, with beta coefficients of −0.25 (*p* = 1.48E‐12) and −0.23 (*p* = 1.18E‐09) with and without age adjustment, respectively. However, in the WIHS cohort, there was no significant association between DNAmTL and HIV (beta coefficient of −0.01; *p* = 6.80E‐01) after adjusting age. Prior to adjusting for age, DNAmTL showed a significant association with HIV (beta coefficient of −0.13; *p* = 2.65E‐03).

**TABLE 2:** Associations between DNAmTL and the phenotypes of interest.

Phenotype	Cohort	Adjust age	Beta	Beta.95CI	*p*	MD
HIV	VACS1	No	−0.23	(−0.30, −0.16)	**1.18E‐09**	−0.37
		Yes	−0.25	(−0.32, −0.18)	**1.48E‐12**	
	WIHS	No	−0.13	(−0.21, −0.05)	**2.65E‐03**	−0.38
		Yes	−0.01	(−0.08, 0.05)	6.80E‐01	
VACS Index	VACS1	No	−0.005	(−0.006, −0.004)	**5.18E‐19**	−0.19
		Yes	−0.002	(−0.003, −0.001)	**2.82E‐05**	
Cancer	VACS	No	−0.07	(−0.10, −0.03)	**1.37E‐04**	−0.07
		Yes	−0.03	(−0.06, 0.00)	5.85E‐02	

*Note*: The self‐reported race and ethnicity, body mass index (BMI), assay sample batch, smoking status, alcohol consumption (natural logarithm of PEth in VACS and number of drinks per week in WIHS), and five cell type proportions (CD4, CD8, NK, B cell, and monocyte) were adjusted. The *p*‐values < 0.05 are shown in bold.

Abbreviation: MD, Mean difference of DNAmTL between cases and controls.

**FIGURE 1:**
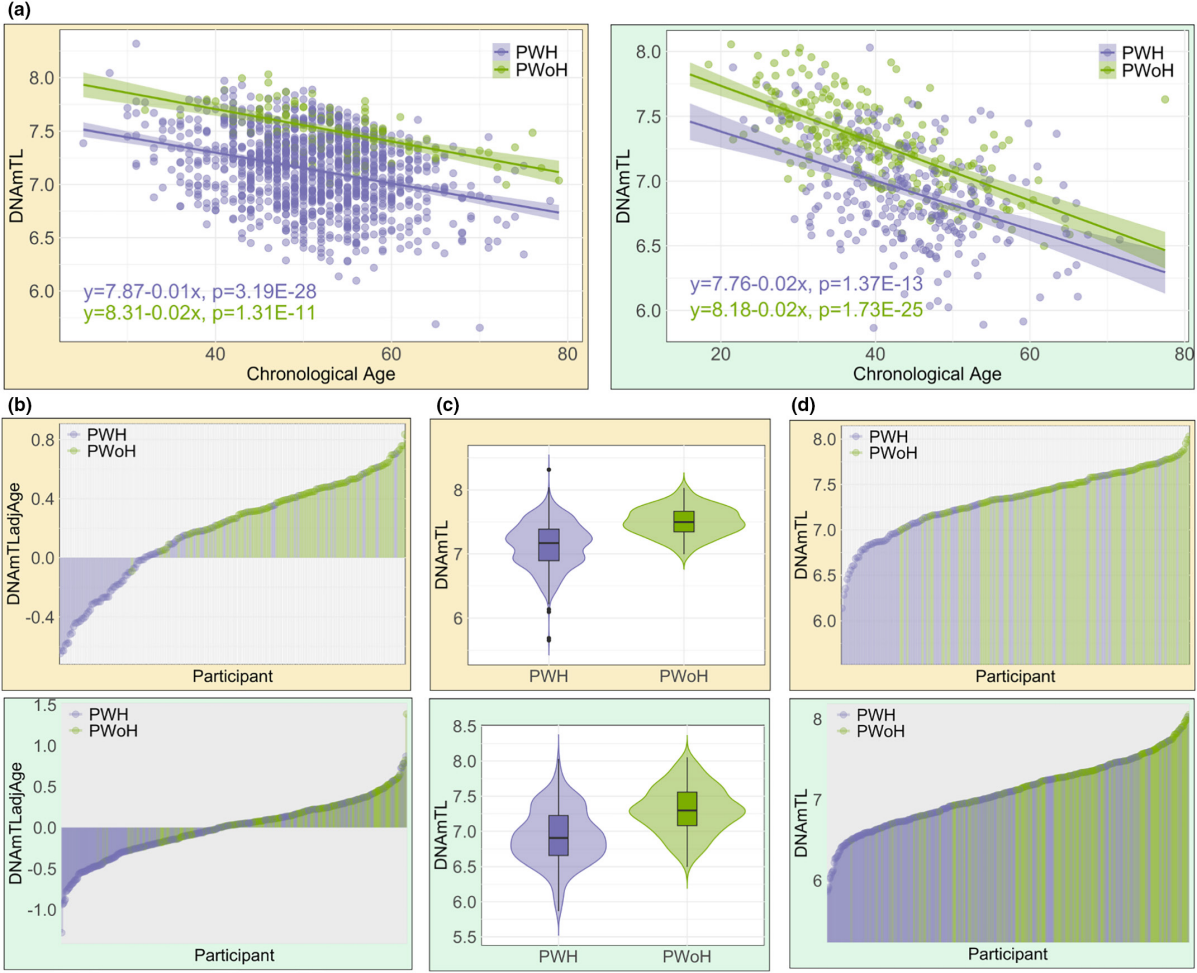
Relationship between DNAmTL and HIV status in Veterans Aging Cohort Study Cohort 1 (VACS1) (yellow background) and Women's Interagency HIV Study (WIHS) (green background). (a) Scatter plot of DNAmTL and chronological age for people with HIV (PWH) and without HIV (PWoH). (b) Lollipop plot of DNAmTLadjAge (the residuals of regressing DNAmTL on chronological age) between PWH and PWoH. (c) Violin plot of DNAmTL between PWH and PWoH. (d) Lollipop plot of DNAmTL between PWH and PWoH.

As shown in Figure 1a, DNAmTL decreased with advancing age in both PWH and PWoH for VACS and WIHS cohorts. To compare two regression lines, we performed the analysis of covariance (ANCOVA) to assess the effect of HIV status on DNAmTL while controlling for the effect of age. The *p*‐value of *F*‐test for the interaction term between age and HIV was 0.85 in VACS1 and 0.31 in WIHS, indicating that the slope of the regression between DNAmTL and age is similar for both PWH and PWoH. However, there is a noticeable distinction between the two groups, with the group of PWH having systematically shorter DNAmTL compared to the group of PWoH (i.e., the regression line being lower on the y‐axis, with the intercepts of regression lines for PWH and PWoH being 7.87 and 8.31 in VACS1, and 7.76 and 8.18 in WIHS, respectively). This positioning suggests that, on average, PWH have shorter DNAmTL compared to those PWoH across the age span. In Figure 1b, we conducted a comparison of DNAmTLadjAge between PWH and PWoH. DNAmTLadjAge captures the unexplained variation in DNAmTL after accounting for the impact of age. Notably, the majority of PWoH displayed a positive DNAmTLadjAge, suggesting that their DNAmTL was longer than expected given their age. In contrast, PWH exhibited a negative DNAmTLadjAge, indicating that their DNAmTL was shorter than expected for their age. As shown in Figure 1c and Figure 1d, in both cohorts, PWH demonstrated a notably shorter average DNAmTL compared to PWoH (mean difference (MD) = −0.37 in VACS1 and MD = −0.38 in WIHS). We first performed the *F*‐test to test the null hypothesis of equal variance in DNAmTL between PWH and PWoH. The results revealed significant differences in variance between the two groups (*p* = 6.00E‐08 in VACS1 and *p* = 0.02 in WIHS). We then conducted a two‐sample *t*‐test with unequal variance to test the mean DNAmTL in PWH and PWoH. The resulting *p*‐values were 3.10E‐32 in VACS1 and 1.43E‐26 in WIHS, indicating a significant difference in mean DNAmTL between the two groups.

We conducted further assessments to examine the relationship between other epigenetic aging clocks and HIV infection across two cohorts (as shown in Table S2). In the VACS1, DNAmMono, DNAmPheno, and DunedinPoAm consistently demonstrated a positive association with HIV, regardless of whether age was adjusted for or not. Specifically, PWH exhibited an average DNAmMono that was notably higher by 3.56 years compared to PWoH (*p* = 1.08E‐04 with adjusting age). Similarly, PWH had an average DNAmPheno higher by 3.71 years compared to PWoH (*p* = 9.23E‐05 with adjusting age), and an average DunedinPoAm higher by 0.06 years compared to PWoH (*p* = 9.07E‐09 with adjusting age). In the WIHS cohort, all epigenetic clocks except DunedinPoAm and EEAA initially showed a significant positive association with HIV when age was not considered. However, when age was taken into account, only DNAmHorvath (*p* = 1.36E‐02) and IEAA (*p* = 7.84E‐03) maintained a significant association with HIV.

We further performed an inverse variance weighted meta‐analysis to combine the results from the two cohorts as outlined in Table S3 (Doi et al., 2015). After adjusting for age, significant associations with HIV were observed for DNAmMono (*p* = 7.02E‐04), DNAmPheno (*p* = 4.34E‐05), DunedinPoAm (*p* = 9.24E‐09), and IEAA (*p* = 7.52E‐03). Following Bonferroni correction, DNAmMono ($p_{\text{Bonf}}$ = 1.26E‐02), DNAmPheno ($p_{\text{Bonf}}$ = 7.82E‐04), and DunedinPoAm ($p_{\mathrm{Bonf}}$ = 1.66E‐07) remained significantly associated with HIV. The DNAmPheno estimates a multifactorial phenotypic age score comprised of nine clinical markers, such as lymphocyte percentage, mean red cell volume, red cell distribution width, albumin, and glucose levels, and chronological age (Bergsma & Rogaeva, 2020; Levine et al., 2018). The markers were selected from the Cox penalized regression model where the hazard of aging‐related mortality was regressed on the clinical markers and chronological age. Similarly, DunedinPoAm estimates Pace of Aging comprised of 18 blood‐chemistry and organ‐system‐function biomarkers such as creatinine clearance, white blood cell count, and HDL cholesterol (Belsky et al., 2020). Notably, studies have shown that the total lymphocyte count appears to be a useful predictor of significant immunosuppression as measured by a CD4+ T‐cell count in PWH (Beck et al., 1996; Blatt et al., 1993).

We extended our investigation to examine the association between DNAmTL and HIV physiologic frailty (VACS Index), focusing solely among PWH in the VACS1 cohort (Table 2). It is worth noting that individuals with higher VACS Index scores typically experience poorer HIV‐related outcomes, and elevated VACS Index scores are indicative of an increased mortality risk (Akgun et al., 2014). As expected, our analysis revealed a negative correlation between DNAmTL and VACS Index. Specifically, when age was included as a covariate in the model, the beta coefficient was −0.002 (*p* = 2.82E‐05). When age was excluded as a covariate, we observed a beta coefficient of −0.005 (*p* = 5.18E‐19).

**FIGURE 2:**
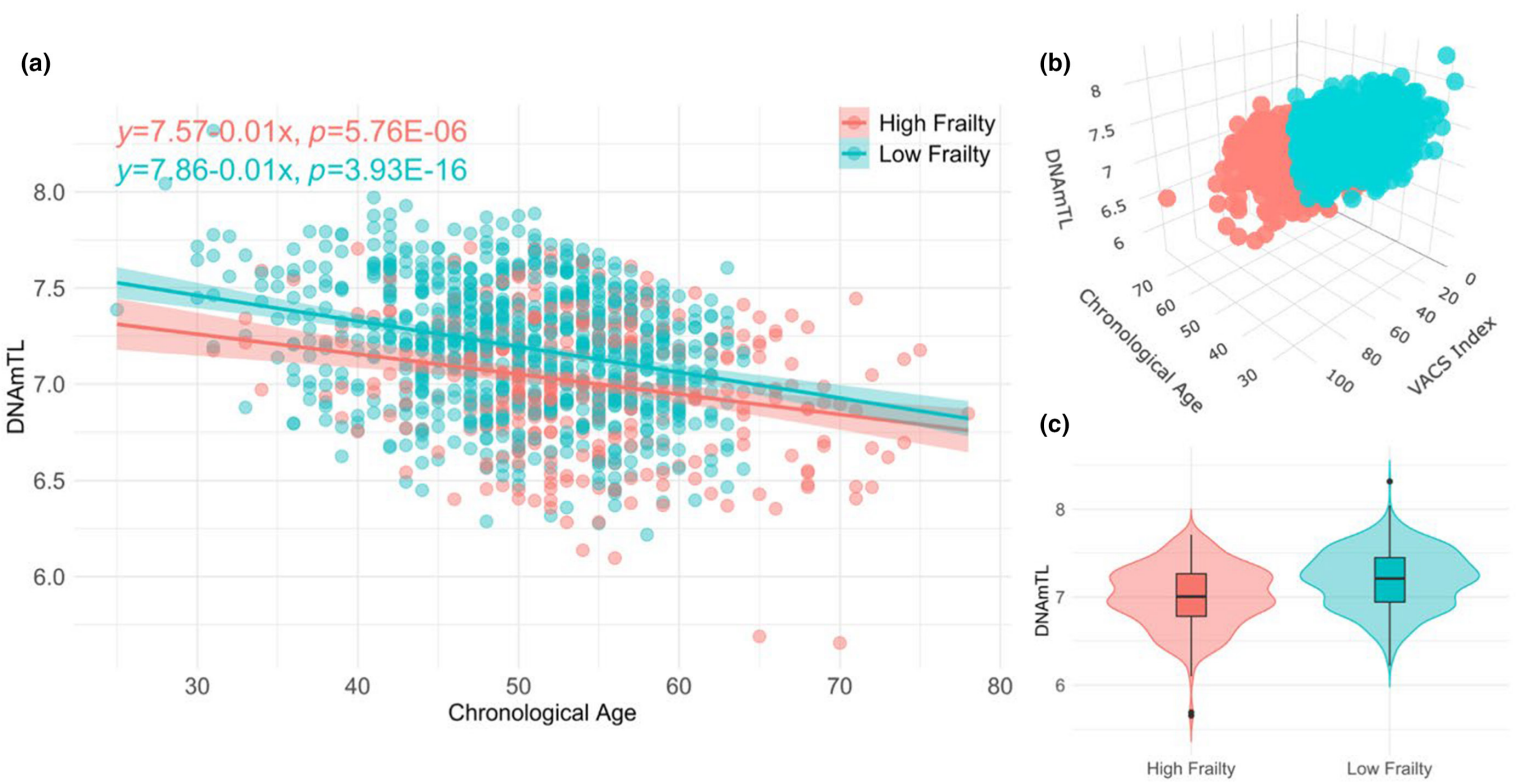
Relationship between DNAmTL and VACS Index in the Veterans Aging Cohort Study Cohort 1 (VACS1). High frailty was defined as VACS Index ≥40, and low frailty was defined as VACS Index <40. (a) Scatter plot of DNAmTL and chronological age for high and low frailty. (b) 3D plot of DNAmTL (z‐axis) and chronological age (x‐axis) for high and low frailty (y‐axis). (c) Violin plot of DNAmTL between high and low frailty.

In Figure 2, we extended our analysis by stratifying the VACS Index into high (VACS Index ≥ 40) and low (VACS Index < 40) frailty. As depicted in Figure 2a,b, DNAmTL exhibited a trend of decreasing with advancing age in all VACS1 samples. Of note, across the age spectrum, PWH with high frailty consistently displayed shorter DNAmTL compared to PWH with low frailty (with the intercepts of regression lines for high frailty and low frailty being 7.57 and 7.86, respectively). To compare two regression lines, we still applied ANCOVA to assess the effect of frailty on DNAmTL while controlling for age. The *p*‐value of *F*‐test for the interaction term between age and frailty was 0.28, indicating that the slope of the regression between DNAmTL and age is similar for both high and low frailty groups. Figure 2c shows the mean difference of DNAmTL between high and low frailty groups was −0.19. The *F*‐test indicated no significant differences in variance between high and low frailty groups (*p* = 0.3). Therefore, we used a two‐sample *t*‐test with equal variance to compare the mean DNAmTL in high and low frailty groups. The resulting *p*‐value was 1.36E‐17, indicating a significant difference in mean DNAmTL between the two groups. These findings collectively underscore the potential relevance of DNAmTL in the context of HIV physiologic frailty, highlighting its association with VACS Index values and its potential as a marker for discerning health disparities within the cohort.

In addition to DNAmTL, we explored the association between different epigenetic aging clocks and the VACS Index (Table S4). DNAmMono, DNAmHorvath, DNAmHannum, DNAmPheno, and DNAmGrim all showed a significant positive association with the VACS Index before adjusting age (beta = 0.143 ~ 0.183, *p* = 3.88E‐35 ~ 6.46E‐19). However, after adding age as a covariate, only DNAmPheno maintained a significant association with VACS Index (beta = 0.048, *p* = 1.43E‐03). Following Bonferroni correction, only DNAmPheno ($p_{\text{Bonf}}$ = 2.58E‐02) remained significantly associated with VACS Index.

### 3.3 Association between shortened DNAmTL and cancer

**FIGURE 3:**
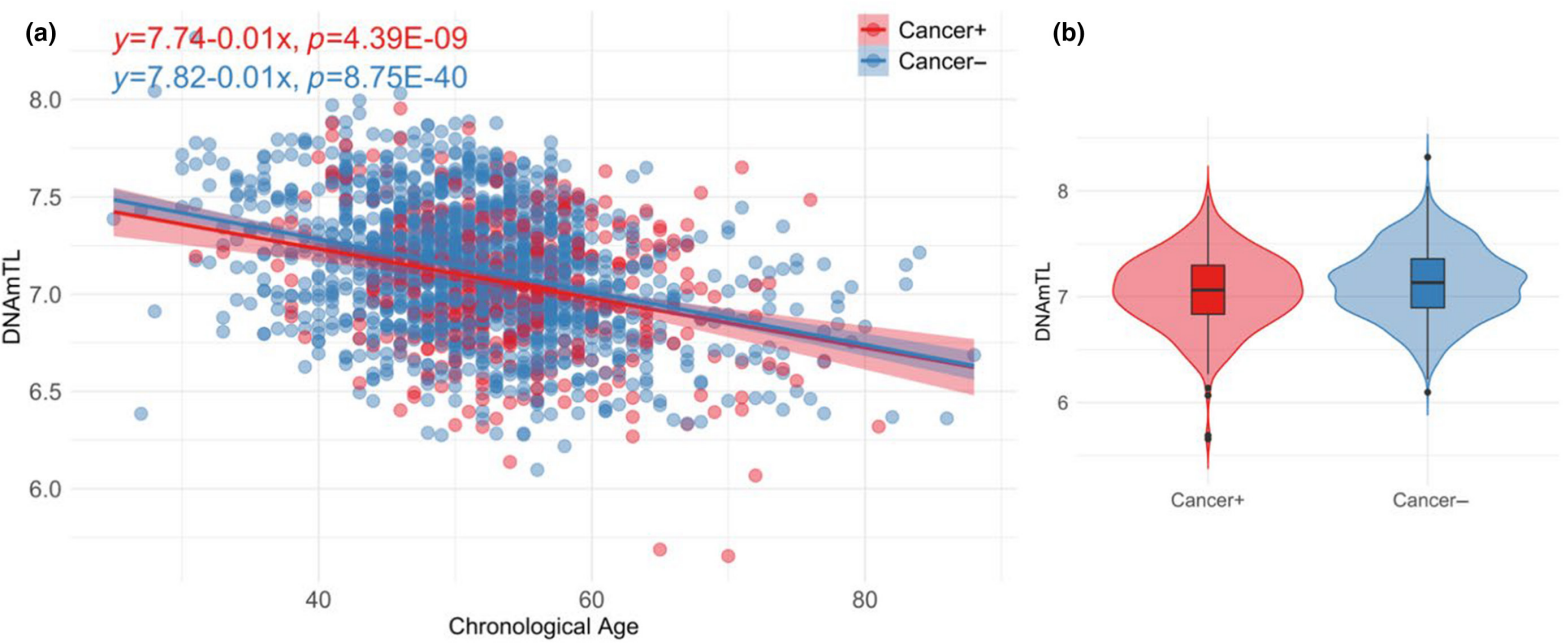
Relationship between DNAmTL and chronological age for cancer cases and controls in Veterans Aging Cohort Study (VACS). (a) Scatter plot of DNAmTL and chronological age for cancer cases (Cancer+) and cancer controls (Cancer‐). (b) Violin plot of DNAmTL between Cancer+ and Cancer‐.

Within the VACS cohort, we conducted an examination of the association between DNAmTL and cancer status (421 cancer+ and 1367 cancer‐), while accounting for various demographic and clinical variables, including self‐reported race and ethnicity, BMI, batch, smoking status, alcohol consumption, and the proportions of five cell types (as shown in Table 2 and Figure 3). Cancer was not associated with DNAmTL when age was included in the model (*p* = 5.85E‐02). However, the analysis revealed a significant negative association between DNAmTL and cancer for the model without age; specifically, the presence of cancer was associated with a reduction of approximately 0.07 kilobases in DNAmTL (*p* = 1.37E‐04).

We observed significant associations between DNAmTL and age in both the cancer+ group (*p* = 4.39E‐09) and the cancer‐ group (*p* = 8.75E‐40) (Figure 3a). Figure 3b indicates a slightly decreased DNAmTL among cancer+ individuals when compared to controls. Specifically, the *F*‐test indicated no significant differences in variance between cancer+ and cancer‐ (*p* = 0.5). Consequently, we applied a two‐sample *t*‐test with equal variance to compare the mean DNAmTL in cancer+ and cancer−. The resulting *p*‐value was 1.96E‐03, indicating a significant difference in mean DNAmTL between the two groups.

Table S5 presents the association between different epigenetic ages and cancer in VACS. Following adjustment for age in the model, DNAmMono and DNAmGrim showed significant associations with cancer with p‐values of 3.07E‐02 and 3.69E‐02, respectively. Specifically, cancer+ had DNAmMono that was 0.93 years older and DNAmGrim that was 0.64 years older compared to cancer−. These findings align with expectations, as monocytes, innate immune cells of the mononuclear phagocyte system, are known to play an important regulatory role in cancer development and progression (Chen et al., 2023; Olingy et al., 2019). Additionally, previous study has demonstrated that DNAmGrim can serve as a mortality risk estimator, particularly notable for its predictive ability for time‐to‐death, time‐to‐coronary heart disease, and time‐to‐cancer (Lu, Quach, et al., 2019). However, after applying Bonferroni correction, DNAmMono ($p_{\text{Bonf}}$ = 5.52E‐01) and DNAmGrim ($p_{\text{Bonf}}$ = 6.65E‐01) were no longer significantly associated with cancer.

### 3.4 DNAmTL shortening and cancer increase risk of all‐cause mortality

**FIGURE 4:**
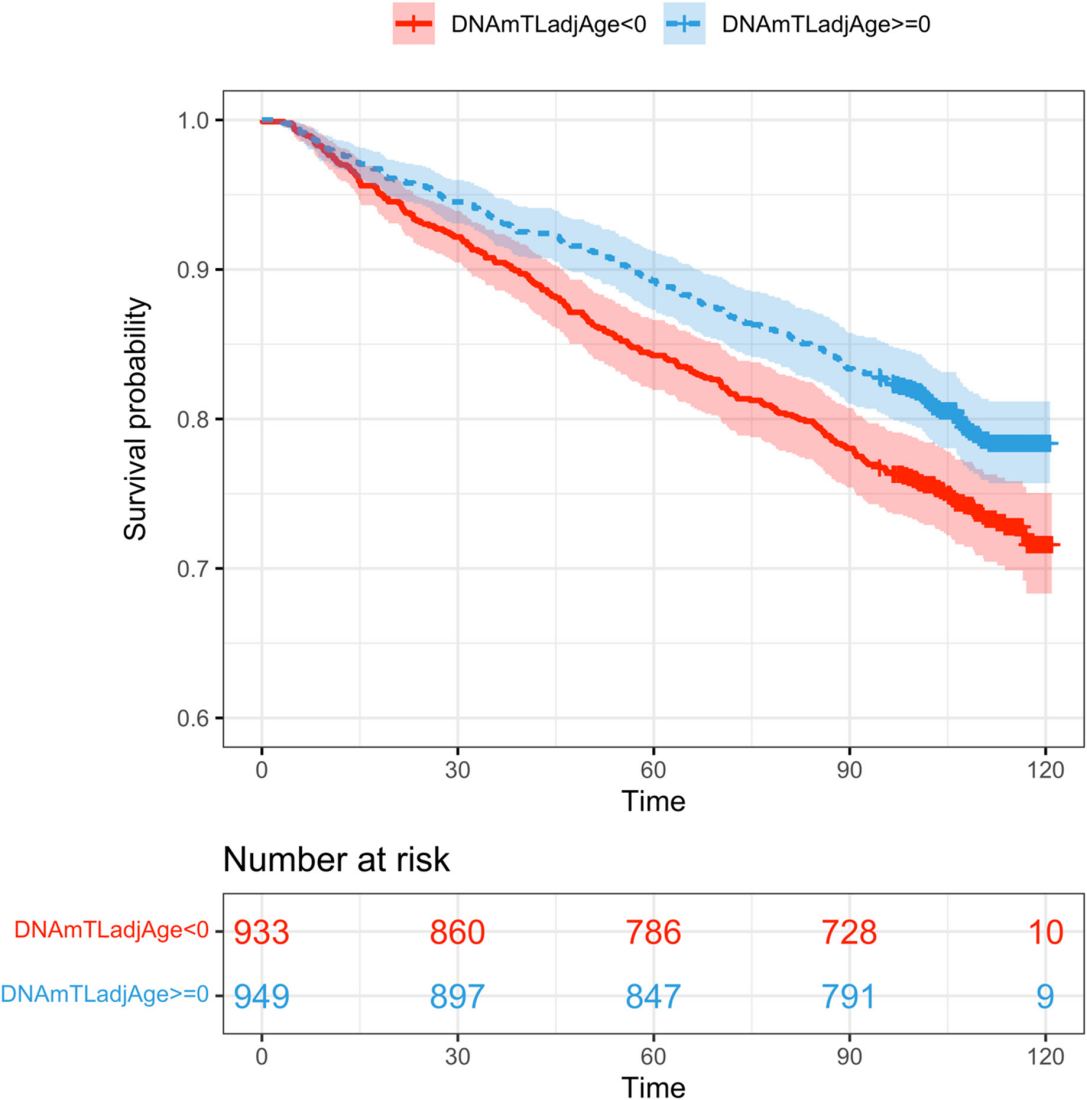
Kaplan–Meier curves of DNAmTLadjAge (the residuals of regressing DNAmTL on chronological age) in Veterans Aging Cohort Study Cohort (VACS), with time measured in months.

We performed the Cox proportional hazards regression analysis to evaluate the potential association between DNAmTL and cancer with mortality rate while accounting for relevant confounding variables (i.e., age, self‐reported race and ethnicity, smoking status, and alcohol consumption) (Table S6). The HR of DNAmTL was 0.60 [95% confidence interval (CI): (0.44, 0.82); *p* = 1.42E‐03], indicating one kilobase decrease in DNAmTL was associated with a 40% increase in mortality risk. Furthermore, Kaplan–Meier curves depicted in Figure 4 showed that the group with a negative DNAmTLadjAge was associated with lower survival probability in comparison with the positive DNAmTLadjAge group. Of note, the HR of cancer on mortality risk was 1.39 [95% CI: (1.12–1.72); *p* = 2.88E‐03], indicating a 39% higher risk of mortality for those with cancer. These findings underscore the potential significance of DNAmTL and cancer status in predicting survival outcomes, highlighting their roles as potential indicators of mortality risk within the studied cohort.

## 4 DISCUSSION

Telomere length serves as a critical indicator of cellular aging and health, influenced by a spectrum of factors including chronic stress, inflammation, lifestyle choices, and specific medical conditions (Starkweather et al., 2014). Its association with various aging‐related diseases underscores its importance as a biomarker of overall health. Applying DNAmTL, we found that HIV infection and cancer diagnoses were negatively associated with DNAmTL, in which PWH and participants with cancer diagnoses showed shorter lengths compared to PWoH and participants without cancer. Interestingly, higher physical frailty was associated with shorter DNAmTL. Finally, we found a shorter DNAmTL predicted a higher all‐cause mortality risk. Together, these findings indicate that DNAmTL is a meaningful biomarker of the aging process among PWH and its comorbid conditions.

One key advantage of DNAmTL, as elucidated in Lu et al. (Lu, Seeboth, et al., 2019), lies in its capacity for precise measurement of methylated CpG sites. The utilization of DNAmTL as a new indicator of biological age offers a unique perspective into the complex interplay of TL dynamics in the context of HIV and cancer. Our study identified associations between DNAmTL and HIV infection, HIV physiologic frailty, as well as cancer, and their impacts on the risk of all‐cause mortality. Adjusting for a range of demographic and clinical variables, we observed a significant inverse relationship between DNAmTL and HIV serostatus and cancer risk. Furthermore, a shorter DNAmTL was associated with greater frailty and mortality risk, reinforcing the notion that TL alterations could serve as an indicator of disease progression.

We found that HIV infection was associated with DNAmTL in two independent cohorts. A negative association of DNAmTL and HIV infection with and without adjusting for age was observed in the VACS1 cohort. The results are consistent with previous findings of the significant positive association between HIV infection and epigenetic age (Horvath et al., 2018; Horvath & Levine, 2015; Liang et al., 2022; Nelson et al., 2017). For example, research has shown that DNAm age in PWH is on average 11.2 years greater than in PWoH (Nelson et al., 2017). Together, these findings align with the concept of accelerated aging among PWH, possibly due to the heightened immune activation and inflammation characteristic of HIV infection (Aberg, 2012).

This result was replicated in the WIHS cohort without adjusting for age. In WIHS, PWH exhibited notably shorter average DNAmTL compared to those PWoH. Mono, Horvath, Hannum, Pheno, and Grim clocks initially exhibited a significant positive association with HIV when age was not considered. However, when age was taken into account, only Horvath clock maintained a significant correlation with HIV. This suggests that while there is an initial association between various epigenetic aging methods and HIV, adjusting for age refines the relationship, with Horvath clock standing out as the most relevant marker in this context. This difference might be attributed to the age difference between the two cohorts. In VACS1, ages ranged from 25 to 88, with a mean of 52.1 and a median of 52. In WIHS, the age range was 18.4 to 77.5, with a mean of 42.2 and a median of 42.1, making the WIHS participants, on average, 9.8 years younger than those in VACS1. Therefore, the effect of DNAmTL in WIHS might not be as strong as observed in VACS1 after considering chronological age.

We conducted further analysis to explore the relationship between DNAmTL and viral load in VACS1 (Table S7). After adjusting for age, DNAmTL exhibited a significant negative correlation with viral load (beta = −0.04, *p* = 4.56E‐06). Additionally, DNAmPheno, DunedinPoAm, and EEAA displayed a positive association with viral load. Particularly, EEAA demonstrated a robust connection with viral load (beta = 0.58, *p* = 4.18E‐04). These findings suggest that viral load may confound the relationship between DNAmTL and the VACS index and all‐cause mortality in our association analysis. Thus, we included viral load as a covariate in the model (Table S8), the association of DNAmTL and the VACS index and cancer remained significant (beta = −0.01, *p* = 1.77E‐18 for the VACS index, and beta = −0.06, *p* = 1.01E‐02 for cancer diagnosis). However, after age adjustment, the VACS Index did not exhibit a significant association with DNAmTL, contrasting with our prior analysis that excluded viral load.

Additionally, the investigation into HIV‐related physiological frailty using the VACS Index revealed a significant adverse correlation between DNAmTL and the VACS Index in both models, whether age was included as a covariate or not. This aligns with previous findings, which indicated that PWH experiencing age acceleration had significantly greater mean VACS Index scores, suggesting a poorer prognosis (Oursler et al., 2023). These results further underscore the potential of DNAmTL as a predictive biomarker for adverse outcomes and mortality risk in the context of PWH.

We also found that individuals with cancer tend to have DNAmTL that are about 0.07 kilobases lower than those without cancer without adjusting for age. We further identified the significant positive association between different epigenetic ages and cancer. This suggests that individuals with higher epigenetic age may be more prone to cancer development or progression (Perna et al., 2016; Yu et al., 2020; Zheng et al., 2016), although further research is needed to determine the underlying mechanisms and causal relationships. Furthermore, the investigation into the association between DNAmTL, cancer, and all‐cause mortality revealed intriguing insights. Previous research has indicated that TL is associated with all‐cause mortality (Cawthon et al., 2003; Wang et al., 2018; Willeit et al., 2010). After adjusting for relevant covariates, we found that lower DNAmTL was associated with an increased risk of mortality. The HR of DNAmTL indicated that for each kilobase decrease in DNAmTL was associated with a 40% increase in mortality risk. Moreover, the HR of cancer indicated a significantly higher risk of mortality for individuals with cancer. These findings highlight the potential clinical relevance of DNAmTL and cancer status as predictors of survival outcomes.

There are several limitations in this study. While our analysis includes survival time from the VACS cohort, it is important to note that other aspects of the study are cross‐sectional. The associations between DNAmTL and cancer, VACS Index, and the risk of mortality observed in the VACS cohort require independent replication to validate their findings. We found an association of DNAmTL with each phenotype of interest but were unable to dissect the causal relationship. Additionally, employing longitudinal data in future research is crucial for better understanding causal or mediation relationships, as the current dataset may not provide the ideal framework for this purpose. The participants in VACS are men while the participants in WIHS are women. Hence, we were unable to investigate the association in a single cohort encompassing both genders. Additionally, the WIHS cohort did not include data on cancer diagnoses and VACS index, which limited our ability to replicate findings within this dataset. Ideally, it would be beneficial to investigate the alignment between the estimated DNAmTL derived from T‐cell subtypes (such as CD4+ and CD8+) and TL measurements obtained through qPCR or TRF‐based assays, which requires DNA isolated from sorted T cells. In the future, we aim to validate the DNAmTL estimator in T‐cell subtypes derived from PBMCs collected from PWH, while also expanding our data collection efforts to include both male and female participants, gather cancer diagnosis information, and construct the VACS index for a more comprehensive analysis.

Our study included both men and women from two population‐based cohorts. The characteristics of the two cohorts differ in some aspects such as sex, age, self‐reported ancestry, and viral load. The participants in VACS are veterans, the majority self‐identify as AAs, and men. The participants in WIHS are women, and half of them self‐identify as AAs, are relatively younger, and self‐report a different profile of substance use compared to the participants in VACS. DNAmTL in VACS is derived from DNAm in whole blood while DNAmTL in WIHS is from DNAm from PBMCs. Despite these distinct demographic and clinical characteristics, the association of DNAmTL and HIV infection is similar in both cohorts, suggesting a generalizability of the findings. It is regrettable that we are unable to replicate the association of DNAmTL with the VACS index and cancer diagnosis in WIHS. We did not adjust for other medical diagnoses such as cardiovascular disease for mortality prediction due to limited statistical power. Future studies should replicate the findings and consider more comprehensive analyses by including additional potential confounding factors.

In summary, our study employed DNAmTL as a measurement to more rigorously examine the interplay of TL dynamics, HIV infection, HIV physiologic frailty, cancer, and all‐cause mortality. The significant influence of HIV infection and cancer on DNAmTL underscores its potential as an indicator of accelerated aging in PWH. DNAmTL is a biomarker of biological age that improves the prediction of disease‐related outcomes and therapeutic interventions.

## AUTHOR CONTRIBUTIONS

XL was responsible for bioinformatics data processing, statistical analyses, and manuscript preparation. BEA contributed to the interpretation of findings and manuscript preparation. KS and MHC contributed to the manuscript preparation. VCM was involved in data interpretation and manuscript preparation. KX and ACJ were responsible for the study design, study protocol, sample preparation, interpretation of findings, and manuscript preparation. All authors read and approved the final manuscript. ACJ provided DNA samples and clinical data and contributed to the study design, study protocol, sample preparation, interpretation of findings, and manuscript preparation.

## FUNDING INFORMATION

The project was supported by the National Institute on Drug Abuse [R03‐DA039745 (Xu), R01‐DA038632 (Xu), R01‐DA047063 (Xu and Aouizerat), R01‐DA047820 (Xu and Aouizerat)] and the Emory Center for AIDS Research [P30‐AI050409 (Marconi)]. COMpAAAS/Veterans Aging Cohort Study, a CHAART Cooperative Agreement, is supported by the National Institutes of Health: National Institute on Alcohol Abuse and Alcoholism (U24‐AA020794, U01‐AA020790, U01‐AA020795, U01‐AA020799; U10‐AA013566‐completed) and in kind by the US Department of Veterans Affairs. Additional grant support from the National Institute on Drug Abuse R01‐DA035616 is also acknowledged.

## CONFLICT OF INTEREST STATEMENT

VCM has received investigator‐initiated research grants (to the institution) and consultation fees (both unrelated to the current work) from Eli Lilly, Bayer, Gilead Sciences, and ViiV. The remaining authors declare that they have no competing interests.

## Supporting information


Appendix S1.

## Data Availability

Demographic variables, clinical variables, and methylation status for the VACS samples are submitted to the GEO dataset (GSE117861) and are available to the public.

## References

[acel14174-bib-0001] Aberg, J. A. (2012). Aging, inflammation, and HIV infection. Topics in Antiviral Medicine, 20(3), 101–105. 22954610 PMC6148943

[acel14174-bib-0002] Aboutara, N. , Jungen, H. , Szewczyk, A. , Muller, A. , & Iwersen‐Bergmann, S. (2023). PEth 16:0/18:1 and 16:0/18:2 after consumption of low doses of alcohol‐a contribution to cutoff discussion. Drug Testing and Analysis, 15(1), 104–114. 10.1002/dta.3376 36181234

[acel14174-bib-0003] Adams, W. L. , Barry, K. L. , & Fleming, M. F. (1996). Screening for problem drinking in older primary care patients. JAMA, 276(24), 1964–1967. 8971065

[acel14174-bib-0004] Adimora, A. A. , Ramirez, C. , Benning, L. , Greenblatt, R. M. , Kempf, M. C. , Tien, P. C. , Kassaye, S. G. , Anastos, K. , Cohen, M. , Minkoff, H. , Wingood, G. , Ofotokun, I. , Fischl, M. A. , & Gange, S. (2018). Cohort profile: The Women's interagency HIV study (WIHS). International Journal of Epidemiology, 47(2), 393–394i. 10.1093/ije/dyy021 29688497 PMC5913596

[acel14174-bib-0005] Akgun, K. M. , Tate, J. P. , Crothers, K. , Crystal, S. , Leaf, D. A. , Womack, J. , Brown, T. T. , Justice, A. C. , Oursler, K. K. , & Oursler, K. K. (2014). An adapted frailty‐related phenotype and the VACS index as predictors of hospitalization and mortality in HIV‐infected and uninfected individuals. Journal of Acquired Immune Deficiency Syndromes, 67(4), 397–404. 10.1097/QAI.0000000000000341 25202921 PMC4213242

[acel14174-bib-0006] Allaire, P. , He, J. , Mayer, J. , Moat, L. , Gerstenberger, P. , Wilhorn, R. , Strutz, S. , Kim, D. S. L. , Zeng, C. , Cox, N. , Shay, J. W. , Denny, J. , Bastarache, L. , Hebbring, S. , & Hebbring, S. (2023). Genetic and clinical determinants of telomere length. Human Genetics and Genomics Advances, 4(3), 100201. 10.1016/j.xhgg.2023.100201 37216007 PMC10199259

[acel14174-bib-0007] Astuti, Y. , Wardhana, A. , Watkins, J. , Wulaningsih, W. , & Network, P. R. (2017). Cigarette smoking and telomere length: A systematic review of 84 studies and meta‐analysis. Environmental Research, 158, 480–489. 10.1016/j.envres.2017.06.038 28704792 PMC5562268

[acel14174-bib-0008] Bacon, M. C. , von Wyl, V. , Alden, C. , Sharp, G. , Robison, E. , Hessol, N. , Gange, S. , Barranday, Y. , Holman, S. , Weber, K. , & Young, M. A. (2005). The Women's interagency HIV study: An observational cohort brings clinical sciences to the bench. Clinical and Diagnostic Laboratory Immunology, 12(9), 1013–1019. 10.1128/CDLI.12.9.1013-1019.2005 16148165 PMC1235804

[acel14174-bib-0009] Beck, E. J. , Kupek, E. J. , Gompels, M. M. , & Pinching, A. J. (1996). Correlation between total and CD4 lymphocyte counts in HIV infection: Not making the good an enemy of the not so perfect. International Journal of STD & AIDS, 7(6), 422–428. 10.1258/0956462961918392 8940671

[acel14174-bib-0010] Belsky, D. W. , Caspi, A. , Arseneault, L. , Baccarelli, A. , Corcoran, D. L. , Gao, X. , Hannon, E. , Harrington, H. L. , Rasmussen, L. J. , Houts, R. , Huffman, K. , Kraus, W. E. , Kwon, D. , Mill, J. , Pieper, C. F. , Prinz, J. A. , Poulton, R. , Schwartz, J. , Sugden, K. , … Moffitt, T. E. (2020). Quantification of the pace of biological aging in humans through a blood test, the DunedinPoAm DNA methylation algorithm. eLife, 9, e54870. 10.7554/eLife.54870 32367804 PMC7282814

[acel14174-bib-0011] Bergsma, T. , & Rogaeva, E. (2020). DNA methylation clocks and their predictive capacity for aging phenotypes and Healthspan. Neuroscience Insights, 15, 2633105520942221. 10.1177/2633105520942221 32743556 PMC7376380

[acel14174-bib-0012] Biver, E. (2022). Osteoporosis and HIV infection. Calcified Tissue International, 110(5), 624–640. 10.1007/s00223-022-00946-4 35098324 PMC9013331

[acel14174-bib-0013] Blasco, M. A. (2007). Telomere length, stem cells and aging. Nature Chemical Biology, 3(10), 640–649. 10.1038/nchembio.2007.38 17876321

[acel14174-bib-0014] Blatt, S. P. , Lucey, C. R. , Butzin, C. A. , Hendrix, C. W. , & Lucey, D. R. (1993). Total lymphocyte count as a predictor of absolute CD4+ count and CD4+ percentage in HIV‐infected persons. JAMA, 269(5), 622–626. 8093628

[acel14174-bib-0015] Broberg, K. , Bjork, J. , Paulsson, K. , Hoglund, M. , & Albin, M. (2005). Constitutional short telomeres are strong genetic susceptibility markers for bladder cancer. Carcinogenesis, 26(7), 1263–1271. 10.1093/carcin/bgi063 15746160

[acel14174-bib-0016] Brown, L. , Needham, B. , & Ailshire, J. (2017). Telomere length among older U.S. adults: Differences by race/ethnicity, gender, and age. Journal of Aging and Health, 29(8), 1350–1366. 10.1177/0898264316661390 27469599 PMC5272874

[acel14174-bib-0017] Calcagno, A. , Nozza, S. , Muss, C. , Celesia, B. M. , Carli, F. , Piconi, S. , De Socio, G. V. , Cattelan, A. M. , Orofino, G. , Ripamonti, D. , Riva, A. , Di Perri, G. , & Di Perri, G. (2015). Ageing with HIV: A multidisciplinary review. Infection, 43(5), 509–522. 10.1007/s15010-015-0795-5 25987480

[acel14174-bib-0018] Capper, R. , Britt‐Compton, B. , Tankimanova, M. , Rowson, J. , Letsolo, B. , Man, S. , Haughton, M. , & Baird, D. M. (2007). The nature of telomere fusion and a definition of the critical telomere length in human cells. Genes & Development, 21(19), 2495–2508. 10.1101/gad.439107 17908935 PMC1993879

[acel14174-bib-0019] Carbone, A. , Vaccher, E. , & Gloghini, A. (2022). Hematologic cancers in individuals infected by HIV. Blood, 139(7), 995–1012. 10.1182/blood.2020005469 34469512

[acel14174-bib-0020] Cawthon, R. M. , Smith, K. R. , O'Brien, E. , Sivatchenko, A. , & Kerber, R. A. (2003). Association between telomere length in blood and mortality in people aged 60 years or older. Lancet, 361(9355), 393–395. 10.1016/S0140-6736(03)12384-7 12573379

[acel14174-bib-0021] Chen, B. H. , Marioni, R. E. , Colicino, E. , Peters, M. J. , Ward‐Caviness, C. K. , Tsai, P. C. , Roetker, N. S. , Just, A. C. , Demerath, E. W. , Guan, W. , Bressler, J. , Fornage, M. , Studenski, S. , Vandiver, A. R. , Moore, A. Z. , Tanaka, T. , Kiel, D. P. , Liang, L. , Vokonas, P. , … Horvath, S. (2016). DNA methylation‐based measures of biological age: Meta‐analysis predicting time to death. Aging (Albany NY), 8(9), 1844–1865. 10.18632/aging.101020 27690265 PMC5076441

[acel14174-bib-0022] Chen, X. , Li, Y. , Xia, H. , & Chen, Y. H. (2023). Monocytes in tumorigenesis and tumor immunotherapy. Cells, 12(13), 1673. 10.3390/cells12131673 37443711 PMC10340267

[acel14174-bib-0023] Chen, Y. A. , Lemire, M. , Choufani, S. , Butcher, D. T. , Grafodatskaya, D. , Zanke, B. W. , Gallinger, S. , Hudson, T. J. , & Weksberg, R. (2013). Discovery of cross‐reactive probes and polymorphic CpGs in the Illumina Infinium HumanMethylation450 microarray. Epigenetics, 8(2), 203–209. 10.4161/epi.23470 23314698 PMC3592906

[acel14174-bib-0024] De Vivo, I. , Prescott, J. , Wong, J. Y. , Kraft, P. , Hankinson, S. E. , & Hunter, D. J. (2009). A prospective study of relative telomere length and postmenopausal breast cancer risk. Cancer Epidemiology, Biomarkers & Prevention, 18(4), 1152–1156. 10.1158/1055-9965.EPI-08-0998 PMC2732000 19293310

[acel14174-bib-0025] Doi, S. A. , Barendregt, J. J. , Khan, S. , Thalib, L. , & Williams, G. M. (2015). Advances in the meta‐analysis of heterogeneous clinical trials I: The inverse variance heterogeneity model. Contemporary Clinical Trials, 45(Pt A), 130–138. 10.1016/j.cct.2015.05.009 26003435

[acel14174-bib-0026] Falutz, J. , Branas, F. , & Erlandson, K. M. (2021). Frailty: The current challenge for aging people with HIV. Current Opinion in HIV and AIDS, 16(3), 133–140. 10.1097/COH.0000000000000677 33833208

[acel14174-bib-0027] Fani, L. , Hilal, S. , Sedaghat, S. , Broer, L. , Licher, S. , Arp, P. P. , van Meurs, J. B. J. , Ikram, M. K. , & Ikram, M. A. (2020). Telomere length and the risk of Alzheimer's disease: The Rotterdam study. Journal of Alzheimer's Disease, 73(2), 707–714. 10.3233/JAD-190759 PMC7029372 31839608

[acel14174-bib-0028] Fasching, C. L. (2018). Telomere length measurement as a clinical biomarker of aging and disease. Critical Reviews in Clinical Laboratory Sciences, 55(7), 443–465. 10.1080/10408363.2018.1504274 30265166

[acel14174-bib-0029] Fortin, J. P. , Triche, T. J., Jr. , & Hansen, K. D. (2017). Preprocessing, normalization and integration of the Illumina HumanMethylationEPIC array with minfi. Bioinformatics, 33(4), 558–560. 10.1093/bioinformatics/btw691 28035024 PMC5408810

[acel14174-bib-0030] Gielen, M. , Hageman, G. J. , Antoniou, E. E. , Nordfjall, K. , Mangino, M. , Balasubramanyam, M. , de Meyer, T. , Hendricks, A. E. , Giltay, E. J. , Hunt, S. C. , Nettleton, J. A. , Salpea, K. D. , Diaz, V. A. , Farzaneh‐Far, R. , Atzmon, G. , Harris, S. E. , Hou, L. , Gilley, D. , Hovatta, I. , … group, T . (2018). Body mass index is negatively associated with telomere length: A collaborative cross‐sectional meta‐analysis of 87 observational studies. The American Journal of Clinical Nutrition, 108(3), 453–475. 10.1093/ajcn/nqy107 30535086 PMC6454526

[acel14174-bib-0031] Hannum, G. , Guinney, J. , Zhao, L. , Zhang, L. , Hughes, G. , Sadda, S. , Klotzle, B. , Bibikova, M. , Fan, J. B. , Gao, Y. , Deconde, R. , Chen, M. , Rajapakse, I. , Friend, S. , Ideker, T. , & Zhang, K. (2013). Genome‐wide methylation profiles reveal quantitative views of human aging rates. Molecular Cell, 49(2), 359–367. 10.1016/j.molcel.2012.10.016 23177740 PMC3780611

[acel14174-bib-0032] Hastings, W. J. , Shalev, I. , & Belsky, D. W. (2017). Translating measures of biological aging to test effectiveness of Geroprotective interventions: What can we learn from research on telomeres? Frontiers in Genetics, 8, 164. 10.3389/fgene.2017.00164 29213278 PMC5702647

[acel14174-bib-0033] Heiss, J. A. , & Just, A. C. (2018). Identifying mislabeled and contaminated DNA methylation microarray data: An extended quality control toolset with examples from GEO. Clinical Epigenetics, 10, 73. 10.1186/s13148-018-0504-1 29881472 PMC5984806

[acel14174-bib-0034] Horvath, S. (2013). DNA methylation age of human tissues and cell types. Genome Biology, 14(10), R115. 10.1186/gb-2013-14-10-r115 24138928 PMC4015143

[acel14174-bib-0035] Horvath, S. , & Levine, A. J. (2015). HIV‐1 infection accelerates age according to the epigenetic clock. The Journal of Infectious Diseases, 212(10), 1563–1573. 10.1093/infdis/jiv277 25969563 PMC4621253

[acel14174-bib-0036] Horvath, S. , & Raj, K. (2018). DNA methylation‐based biomarkers and the epigenetic clock theory of ageing. Nature Reviews. Genetics, 19(6), 371–384. 10.1038/s41576-018-0004-3 29643443

[acel14174-bib-0037] Horvath, S. , Stein, D. J. , Phillips, N. , Heany, S. J. , Kobor, M. S. , Lin, D. T. S. , Myer, L. , Zar, H. J. , Levine, A. J. , & Hoare, J. (2018). Perinatally acquired HIV infection accelerates epigenetic aging in south African adolescents. AIDS, 32(11), 1465–1474. 10.1097/QAD.0000000000001854 29746298 PMC6026068

[acel14174-bib-0038] Houseman, E. A. , Accomando, W. P. , Koestler, D. C. , Christensen, B. C. , Marsit, C. J. , Nelson, H. H. , Wiencke, J. K. , & Kelsey, K. T. (2012). DNA methylation arrays as surrogate measures of cell mixture distribution. BMC Bioinformatics, 13, 86. 10.1186/1471-2105-13-86 22568884 PMC3532182

[acel14174-bib-0039] Hu, H. , Li, B. , & Duan, S. (2018). The alteration of subtelomeric DNA methylation in aging‐related diseases. Frontiers in Genetics, 9, 697. 10.3389/fgene.2018.00697 30687384 PMC6333653

[acel14174-bib-0040] Jang, J. S. , Choi, Y. Y. , Lee, W. K. , Choi, J. E. , Cha, S. I. , Kim, Y. J. , Kim, C. H. , Kam, S. , Jung, T. H. , & Park, J. Y. (2008). Telomere length and the risk of lung cancer. Cancer Science, 99(7), 1385–1389. 10.1111/j.1349-7006.2008.00831.x 18452563 PMC11158548

[acel14174-bib-0041] Jung, J. , McCartney, D. L. , Wagner, J. , Rosoff, D. B. , Schwandt, M. , Sun, H. , Wiers, C. E. , de Carvalho, L. M. , Volkow, N. D. , Walker, R. M. , Campbell, A. , Porteous, D. J. , McIntosh, A. M. , Marioni, R. E. , Horvath, S. , Evans, K. L. , & Lohoff, F. W. (2022). Alcohol use disorder is associated with DNA methylation‐based shortening of telomere length and regulated by TESPA1: Implications for aging. Molecular Psychiatry, 27(9), 3875–3884. 10.1038/s41380-022-01624-5 35705636 PMC9708583

[acel14174-bib-0042] Justice, A. C. , Dombrowski, E. , Conigliaro, J. , Fultz, S. L. , Gibson, D. , Madenwald, T. , Goulet, J. , Simberkoff, M. , Butt, A. A. , Rimland, D. , Rodriguez‐Barradas, M. C. , Gibert, C. L. , Oursler, K. A. , Brown, S. , Leaf, D. A. , Goetz, M. B. , & Bryant, K. (2006). Veterans aging cohort study (VACS): Overview and description. Medical Care, 44(8 Suppl 2), S13–S24. 10.1097/01.mlr.0000223741.02074.66 16849964 PMC3049942

[acel14174-bib-0043] Justice, A. C. , Freiberg, M. S. , Tracy, R. , Kuller, L. , Tate, J. P. , Goetz, M. B. , Fiellin, D. A. , Vanasse, G. J. , Butt, A. A. , Rodriguez‐Barradas, M. C. , Gibert, C. , Oursler, K. A. , Deeks, S. G. , Bryant, K. , & Team, V. P . (2012). Does an index composed of clinical data reflect effects of inflammation, coagulation, and monocyte activation on mortality among those aging with HIV? Clinical Infectious Diseases, 54(7), 984–994. 10.1093/cid/cir989 22337823 PMC3297653

[acel14174-bib-0044] Justice, A. C. , Modur, S. P. , Tate, J. P. , Althoff, K. N. , Jacobson, L. P. , Gebo, K. A. , Kitahata, M. M. , Horberg, M. A. , Brooks, J. T. , Buchacz, K. , Rourke, S. B. , Rachlis, A. , Napravnik, S. , Eron, J. , Willig, J. H. , Moore, R. , Kirk, G. D. , Bosch, R. , Rodriguez, B. , … Teams, V. P . (2013). Predictive accuracy of the veterans aging cohort study index for mortality with HIV infection: A north American cross cohort analysis. Journal of Acquired Immune Deficiency Syndromes, 62(2), 149–163. 10.1097/QAI.0b013e31827df36c 23187941 PMC3619393

[acel14174-bib-0045] Jylhava, J. , Pedersen, N. L. , & Hagg, S. (2017). Biological age predictors. eBioMedicine, 21, 29–36. 10.1016/j.ebiom.2017.03.046 28396265 PMC5514388

[acel14174-bib-0046] Khosravaniardakani, S. , Bokov, D. O. , Mahmudiono, T. , Hashemi, S. S. , Nikrad, N. , Rabieemotmaen, S. , & Abbasalizad‐Farhangi, M. (2022). Obesity accelerates leukocyte telomere length shortening in apparently healthy adults: A meta‐analysis. Frontiers in Nutrition, 9, 812846. 10.3389/fnut.2022.812846 35719148 PMC9199514

[acel14174-bib-0047] Kimura, M. , Stone, R. C. , Hunt, S. C. , Skurnick, J. , Lu, X. , Cao, X. , Harley, C. B. , & Aviv, A. (2010). Measurement of telomere length by the southern blot analysis of terminal restriction fragment lengths. Nature Protocols, 5(9), 1596–1607. 10.1038/nprot.2010.124 21085125

[acel14174-bib-0048] Lai, T. P. , Wright, W. E. , & Shay, J. W. (2018). Comparison of telomere length measurement methods. Philosophical Transactions of the Royal Society of London. Series B, Biological Sciences, 373(1741), 20160451. 10.1098/rstb.2016.0451 29335378 PMC5784071

[acel14174-bib-0049] Lan, Q. , Cawthon, R. , Shen, M. , Weinstein, S. J. , Virtamo, J. , Lim, U. , Hosgood, H. D., 3rd , Albanes, D. , Rothman, N. , & Rothman, N. (2009). A prospective study of telomere length measured by monochrome multiplex quantitative PCR and risk of non‐Hodgkin lymphoma. Clinical Cancer Research, 15(23), 7429–7433. 10.1158/1078-0432.CCR-09-0845 19934287 PMC2787641

[acel14174-bib-0050] Latifovic, L. , Peacock, S. D. , Massey, T. E. , & King, W. D. (2016). The influence of alcohol consumption, cigarette smoking, and physical activity on leukocyte telomere length. Cancer Epidemiology, Biomarkers & Prevention, 25(2), 374–380. 10.1158/1055-9965.EPI-14-1364 26656293

[acel14174-bib-0051] Lee, I. M. , Lin, J. , Castonguay, A. J. , Barton, N. S. , Buring, J. E. , & Zee, R. Y. (2010). Mean leukocyte telomere length and risk of incident colorectal carcinoma in women: A prospective, nested case‐control study. Clinical Chemistry and Laboratory Medicine, 48(2), 259–262. 10.1515/CCLM.2010.049 19961392 PMC2818287

[acel14174-bib-0052] Lehne, B. , Drong, A. W. , Loh, M. , Zhang, W. , Scott, W. R. , Tan, S. T. , Afzal, U. , Scott, J. , Jarvelin, M. R. , Elliott, P. , McCarthy, M. I. , Kooner, J. S. , Chambers, J. C. , & Elliott, P. (2015). A coherent approach for analysis of the Illumina HumanMethylation450 BeadChip improves data quality and performance in epigenome‐wide association studies. Genome Biology, 16(1), 37. 25853392 10.1186/s13059-015-0600-x PMC4365767

[acel14174-bib-0053] Levine, M. E. , Lu, A. T. , Quach, A. , Chen, B. H. , Assimes, T. L. , Bandinelli, S. , Hou, L. , Baccarelli, A. A. , Stewart, J. D. , Li, Y. , Whitsel, E. A. , Wilson, J. G. , Reiner, A. P. , Aviv, A. , Lohman, K. , Liu, Y. , Ferrucci, L. , & Horvath, S. (2018). An epigenetic biomarker of aging for lifespan and healthspan. Aging (Albany NY), 10(4), 573–591. 10.18632/aging.101414 29676998 PMC5940111

[acel14174-bib-0054] Liang, X. , Justice, A. C. , So‐Armah, K. , Krystal, J. H. , Sinha, R. , & Xu, K. (2020). DNA methylation signature on phosphatidylethanol, not on self‐reported alcohol consumption, predicts hazardous alcohol consumption in two distinct populations. Molecular Psychiatry, 26, 2238–2253. 10.1038/s41380-020-0668-x 32034291 PMC8440221

[acel14174-bib-0055] Liang, X. , Sinha, R. , Justice, A. C. , Cohen, M. H. , Aouizerat, B. E. , & Xu, K. (2022). A new monocyte epigenetic clock reveals nonlinear effects of alcohol consumption on biological aging in three independent cohorts (N = 2242). Alcoholism, Clinical and Experimental Research, 46(5), 736–748. 10.1111/acer.14803 35257385 PMC9117474

[acel14174-bib-0056] Lu, A. T. , Quach, A. , Wilson, J. G. , Reiner, A. P. , Aviv, A. , Raj, K. , Hou, L. , Baccarelli, A. A. , Li, Y. , Stewart, J. D. , Whitsel, E. A. , Assimes, T. L. , Ferrucci, L. , & Horvath, S. (2019). DNA methylation GrimAge strongly predicts lifespan and healthspan. Aging (Albany NY), 11(2), 303–327. 10.18632/aging.101684 30669119 PMC6366976

[acel14174-bib-0057] Lu, A. T. , Seeboth, A. , Tsai, P. C. , Sun, D. , Quach, A. , Reiner, A. P. , Kooperberg, C. , Ferrucci, L. , Hou, L. , Baccarelli, A. A. , Li, Y. , Harris, S. E. , Corley, J. , Taylor, A. , Deary, I. J. , Stewart, J. D. , Whitsel, E. A. , Assimes, T. L. , Chen, W. , … Horvath, S. (2019). DNA methylation‐based estimator of telomere length. Aging (Albany NY), 11(16), 5895–5923. 10.18632/aging.102173 31422385 PMC6738410

[acel14174-bib-0058] Ma, H. , Zhou, Z. , Wei, S. , Liu, Z. , Pooley, K. A. , Dunning, A. M. , Svenson, U. , Roos, G. , Hosgood, H. D., 3rd , Shen, M. , & Wei, Q. (2011). Shortened telomere length is associated with increased risk of cancer: A meta‐analysis. PLoS One, 6(6), e20466. 10.1371/journal.pone.0020466 21695195 PMC3112149

[acel14174-bib-0059] Maciejowski, J. , & de Lange, T. (2017). Telomeres in cancer: Tumour suppression and genome instability. Nature Reviews. Molecular Cell Biology, 18(3), 175–186. 10.1038/nrm.2016.171 28096526 PMC5589191

[acel14174-bib-0060] Maciejowski, J. , & de Lange, T. (2019). Author correction: Telomeres in cancer: Tumour suppression and genome instability. Nature Reviews. Molecular Cell Biology, 20(4), 259. 10.1038/s41580-019-0113-7 30816301

[acel14174-bib-0061] Maciel, R. A. , Kluck, H. M. , Durand, M. , & Sprinz, E. (2018). Comorbidity is more common and occurs earlier in persons living with HIV than in HIV‐uninfected matched controls, aged 50 years and older: A cross‐sectional study. International Journal of Infectious Diseases, 70, 30–35. 10.1016/j.ijid.2018.02.009 29476902

[acel14174-bib-0062] Mai, S. , & Garini, Y. (2006). The significance of telomeric aggregates in the interphase nuclei of tumor cells. Journal of Cellular Biochemistry, 97(5), 904–915. 10.1002/jcb.20760 16408280

[acel14174-bib-0063] Marcus, J. L. , Leyden, W. A. , Alexeeff, S. E. , Anderson, A. N. , Hechter, R. C. , Hu, H. , Lam, J. O. , Towner, W. J. , Yuan, Q. , Horberg, M. A. , & Silverberg, M. J. (2020). Comparison of overall and comorbidity‐free life expectancy between insured adults with and without HIV infection, 2000‐2016. JAMA Network Open, 3(6), e207954. 10.1001/jamanetworkopen.2020.7954 32539152 PMC7296391

[acel14174-bib-0064] McArthur, J. C. (2004). HIV dementia: an evolving disease. Journal of Neuroimmunology, 157(1–2), 3–10. 10.1016/j.jneuroim.2004.08.042 15579274

[acel14174-bib-0065] McGrath, M. , Wong, J. Y. , Michaud, D. , Hunter, D. J. , & De Vivo, I. (2007). Telomere length, cigarette smoking, and bladder cancer risk in men and women. Cancer Epidemiology, Biomarkers & Prevention, 16(4), 815–819. 10.1158/1055-9965.EPI-06-0961 17416776

[acel14174-bib-0066] Mender, I. , & Shay, J. W. (2015). Telomere restriction fragment (TRF) analysis. Bio‐Protocol, 5(22), e1658. 10.21769/bioprotoc.1658 27500189 PMC4972328

[acel14174-bib-0067] Montpetit, A. J. , Alhareeri, A. A. , Montpetit, M. , Starkweather, A. R. , Elmore, L. W. , Filler, K. , Mohanraj, L. , Burton, C. W. , Menzies, V. S. , Lyon, D. E. , Jackson‐Cook, C. K. , & Jackson‐Cook, C. K. (2014). Telomere length: A review of methods for measurement. Nursing Research, 63(4), 289–299. 10.1097/NNR.0000000000000037 24977726 PMC4292845

[acel14174-bib-0068] Moore, D. J. , Sun‐Suslow, N. , Nichol, A. A. , Paolillo, E. W. , Saloner, R. , Letendre, S. L. , Iudicello, J. , & Morgan, E. E. (2023). The clinical utility of three frailty measures in identifying HIV‐associated neurocognitive disorders. AIDS, 38, 645–655. 10.1097/QAD.0000000000003805 38051787 PMC10939888

[acel14174-bib-0069] Muller, D. , & Gyorffy, B. (2022). DNA methylation‐based diagnostic, prognostic, and predictive biomarkers in colorectal cancer. Biochimica Et Biophysica Acta. Reviews on Cancer, 1877(3), 188722. 10.1016/j.bbcan.2022.188722 35307512

[acel14174-bib-0070] Nelson, K. N. , Hui, Q. , Rimland, D. , Xu, K. , Freiberg, M. S. , Justice, A. C. , Marconi, V. C. , & Sun, Y. V. (2017). Identification of HIV infection‐related DNA methylation sites and advanced epigenetic aging in HIV‐positive, treatment‐naive U.S. veterans. AIDS, 31(4), 571–575. 10.1097/QAD.0000000000001360 27922854 PMC5263111

[acel14174-bib-0071] Noubissi, E. C. , Katte, J. C. , & Sobngwi, E. (2018). Diabetes and HIV. Current Diabetes Reports, 18(11), 125. 10.1007/s11892-018-1076-3 30294763

[acel14174-bib-0072] Nussey, D. H. , Baird, D. , Barrett, E. , Boner, W. , Fairlie, J. , Gemmell, N. , Hartmann, N. , Horn, T. , Haussmann, M. , Olsson, M. , Turbill, C. , Verhulst, S. , Zahn, S. , & Monaghan, P. (2014). Measuring telomere length and telomere dynamics in evolutionary biology and ecology. Methods in Ecology and Evolution, 5(4), 299–310. 10.1111/2041-210X.12161 25834722 PMC4375921

[acel14174-bib-0073] Okazaki, S. , Numata, S. , Otsuka, I. , Horai, T. , Kinoshita, M. , Sora, I. , Ohmori, T. , & Hishimoto, A. (2020). Decelerated epigenetic aging associated with mood stabilizers in the blood of patients with bipolar disorder. Translational Psychiatry, 10(1), 129. 10.1038/s41398-020-0813-y 32366819 PMC7198548

[acel14174-bib-0074] Olingy, C. E. , Dinh, H. Q. , & Hedrick, C. C. (2019). Monocyte heterogeneity and functions in cancer. Journal of Leukocyte Biology, 106(2), 309–322. 10.1002/JLB.4RI0818-311R 30776148 PMC6658332

[acel14174-bib-0075] Oursler, K. K. , Marconi, V. C. , Wang, Z. , Xu, K. , Montano, M. , So‐Armah, K. , Justice, A. C. , & Sun, Y. V. (2023). Epigenetic age acceleration markers are associated with physiologic frailty and all‐cause mortality in people with human immunodeficiency virus. Clinical Infectious Diseases, 76(3), e638–e644. 10.1093/cid/ciac656 35970820 PMC10169393

[acel14174-bib-0076] Pathai, S. , Lawn, S. D. , Gilbert, C. E. , McGuinness, D. , McGlynn, L. , Weiss, H. A. , Port, J. , Christ, T. , Barclay, K. , Wood, R. , Bekker, L. G. , & Shiels, P. G. (2013). Accelerated biological ageing in HIV‐infected individuals in South Africa: A case‐control study. AIDS, 27(15), 2375–2384. 10.1097/QAD.0b013e328363bf7f 23751258 PMC3805356

[acel14174-bib-0077] Pearce, E. E. , Horvath, S. , Katta, S. , Dagnall, C. , Aubert, G. , Hicks, B. D. , Spellman, S. R. , Katki, H. , Savage, S. A. , Alsaggaf, R. , & Gadalla, S. M. (2021). DNA‐methylation‐based telomere length estimator: Comparisons with measurements from flow FISH and qPCR. Aging (Albany NY), 13(11), 14675–14686. 10.18632/aging.203126 34083495 PMC8221337

[acel14174-bib-0078] Perna, L. , Zhang, Y. , Mons, U. , Holleczek, B. , Saum, K. U. , & Brenner, H. (2016). Epigenetic age acceleration predicts cancer, cardiovascular, and all‐cause mortality in a German case cohort. Clinical Epigenetics, 8, 64. 10.1186/s13148-016-0228-z 27274774 PMC4891876

[acel14174-bib-0079] Piano, M. R. , Tiwari, S. , Nevoral, L. , & Phillips, S. A. (2015). Phosphatidylethanol levels are elevated and correlate strongly with AUDIT scores in Young adult binge drinkers. Alcohol and Alcoholism, 50(5), 519–525. 10.1093/alcalc/agv049 26051989

[acel14174-bib-0080] Pusceddu, I. , Kleber, M. , Delgado, G. , Herrmann, W. , Marz, W. , & Herrmann, M. (2018). Telomere length and mortality in the Ludwigshafen risk and cardiovascular health study. PLoS One, 13(6), e0198373. 10.1371/journal.pone.0198373 29920523 PMC6007915

[acel14174-bib-0081] Rewak, M. , Buka, S. , Prescott, J. , De Vivo, I. , Loucks, E. B. , Kawachi, I. , Non, A. L. , & Kubzansky, L. D. (2014). Race‐related health disparities and biological aging: Does rate of telomere shortening differ across blacks and whites? Biological Psychology, 99, 92–99. 10.1016/j.biopsycho.2014.03.007 24686071 PMC4610356

[acel14174-bib-0082] Rockwood, K. , & Mitnitski, A. (2007). Frailty in relation to the accumulation of deficits. The Journals of Gerontology. Series A, Biological Sciences and Medical Sciences, 62(7), 722–727. 10.1093/gerona/62.7.722 17634318

[acel14174-bib-0083] Rockwood, K. , Song, X. , MacKnight, C. , Bergman, H. , Hogan, D. B. , McDowell, I. , & Mitnitski, A. (2005). A global clinical measure of fitness and frailty in elderly people. CMAJ, 173(5), 489–495. 10.1503/cmaj.050051 16129869 PMC1188185

[acel14174-bib-0084] Roomaney, R. A. , van Wyk, B. , & Pillay‐van Wyk, V. (2022). Aging with HIV: Increased risk of HIV comorbidities in older adults. International Journal of Environmental Research and Public Health, 19(4), 2359. 10.3390/ijerph19042359 35206544 PMC8872228

[acel14174-bib-0085] Rossiello, F. , Jurk, D. , Passos, J. F. , & d'Adda di Fagagna, F. (2022). Telomere dysfunction in ageing and age‐related diseases. Nature Cell Biology, 24(2), 135–147. 10.1038/s41556-022-00842-x 35165420 PMC8985209

[acel14174-bib-0086] Russo, A. , Modica, F. , Guarrera, S. , Fiorito, G. , Pardini, B. , Viberti, C. , Allione, A. , Critelli, R. , Bosio, A. , Casetta, G. , Cucchiarale, G. , Destefanis, P. , Gontero, P. , Rolle, L. , Zitella, A. , Fontana, D. , Frea, B. , Vineis, P. , Sacerdote, C. , & Matullo, G. (2014). Shorter leukocyte telomere length is independently associated with poor survival in patients with bladder cancer. Cancer Epidemiology, Biomarkers & Prevention, 23(11), 2439–2446. 10.1158/1055-9965.EPI-14-0228 25234236

[acel14174-bib-0087] Salpea, K. D. , Talmud, P. J. , Cooper, J. A. , Maubaret, C. G. , Stephens, J. W. , Abelak, K. , & Humphries, S. E. (2010). Association of telomere length with type 2 diabetes, oxidative stress and UCP2 gene variation. Atherosclerosis, 209(1), 42–50. 10.1016/j.atherosclerosis.2009.09.070 19889414 PMC2839074

[acel14174-bib-0088] Shao, L. , Wood, C. G. , Zhang, D. , Tannir, N. M. , Matin, S. , Dinney, C. P. , & Wu, X. (2007). Telomere dysfunction in peripheral lymphocytes as a potential predisposition factor for renal cancer. The Journal of Urology, 178(4 Pt 1), 1492–1496. 10.1016/j.juro.2007.05.112 17707063

[acel14174-bib-0089] Shen, J. , Gammon, M. D. , Terry, M. B. , Wang, Q. , Bradshaw, P. , Teitelbaum, S. L. , Neugut, A. I. , & Santella, R. M. (2009). Telomere length, oxidative damage, antioxidants and breast cancer risk. International Journal of Cancer, 124(7), 1637–1643. 10.1002/ijc.24105 19089916 PMC2727686

[acel14174-bib-0090] Shinko, Y. , Okazaki, S. , Otsuka, I. , Horai, T. , Kim, S. , Tanifuji, T. , & Hishimoto, A. (2022). Accelerated epigenetic age and shortened telomere length based on DNA methylation in Nicolaides‐Baraitser syndrome. Molecular Genetics & Genomic Medicine, 10(3), e1876. 10.1002/mgg3.1876 35092358 PMC8922957

[acel14174-bib-0091] Smith, J. A. , Raisky, J. , Ratliff, S. M. , Liu, J. , Kardia, S. L. R. , Turner, S. T. , Mosley, T. H. , & Zhao, W. (2019). Intrinsic and extrinsic epigenetic age acceleration are associated with hypertensive target organ damage in older African Americans. BMC Medical Genomics, 12(1), 141. 10.1186/s12920-019-0585-5 31640709 PMC6806502

[acel14174-bib-0092] So‐Armah, K. , Benjamin, L. A. , Bloomfield, G. S. , Feinstein, M. J. , Hsue, P. , Njuguna, B. , & Freiberg, M. S. (2020). HIV and cardiovascular disease. Lancet HIV, 7(4), e279–e293. 10.1016/S2352-3018(20)30036-9 32243826 PMC9346572

[acel14174-bib-0093] Starkweather, A. R. , Alhaeeri, A. A. , Montpetit, A. , Brumelle, J. , Filler, K. , Montpetit, M. , Mohanraj, L. , Lyon, D. E. , & Jackson‐Cook, C. K. (2014). An integrative review of factors associated with telomere length and implications for biobehavioral research. Nursing Research, 63(1), 36–50. 10.1097/NNR.0000000000000009 24335912 PMC4112289

[acel14174-bib-0094] Stewart, S. H. , Reuben, A. , Brzezinski, W. A. , Koch, D. G. , Basile, J. , Randall, P. K. , & Miller, P. M. (2009). Preliminary evaluation of phosphatidylethanol and alcohol consumption in patients with liver disease and hypertension. Alcohol and Alcoholism, 44(5), 464–467. 19535495 10.1093/alcalc/agp039 PMC2765354

[acel14174-bib-0095] Tate, J. P. , Justice, A. C. , Hughes, M. D. , Bonnet, F. , Reiss, P. , Mocroft, A. , Nattermann, J. , Lampe, F. C. , Bucher, H. C. , Sterling, T. R. , Crane, H. M. , Kitahata, M. M. , May, M. , & Sterne, J. A. (2013). An internationally generalizable risk index for mortality after one year of antiretroviral therapy. AIDS, 27(4), 563–572. 10.1097/QAD.0b013e32835b8c7f 23095314 PMC4283204

[acel14174-bib-0096] Thomas, M. D. , Sohail, S. , Mendez, R. M. , Marquez‐Magana, L. , & Allen, A. M. (2021). Racial discrimination and telomere length in midlife African American women: Interactions of educational attainment and employment status. Annals of Behavioral Medicine, 55(7), 601–611. 10.1093/abm/kaaa104 33289498 PMC8240134

[acel14174-bib-0097] Vaiserman, A. , & Krasnienkov, D. (2020). Telomere length as a marker of biological age: State‐of‐the‐art, open issues, and future perspectives. Frontiers in Genetics, 11, 630186. 10.3389/fgene.2020.630186 33552142 PMC7859450

[acel14174-bib-0098] Valdes, A. M. , Andrew, T. , Gardner, J. P. , Kimura, M. , Oelsner, E. , Cherkas, L. F. , Aviv, A. , & Spector, T. D. (2005). Obesity, cigarette smoking, and telomere length in women. Lancet, 366(9486), 662–664. 10.1016/S0140-6736(05)66630-5 16112303

[acel14174-bib-0099] Viel, G. , Boscolo‐Berto, R. , Cecchetto, G. , Fais, P. , Nalesso, A. , & Ferrara, S. D. (2012). Phosphatidylethanol in blood as a marker of chronic alcohol use: A systematic review and meta‐analysis. International Journal of Molecular Sciences, 13(11), 14788–14812. 10.3390/ijms131114788 23203094 PMC3509610

[acel14174-bib-0100] Wang, Q. , Zhan, Y. , Pedersen, N. L. , Fang, F. , & Hagg, S. (2018). Telomere length and all‐cause mortality: A meta‐analysis. Ageing Research Reviews, 48, 11–20. 10.1016/j.arr.2018.09.002 30254001

[acel14174-bib-0101] White, M. C. , Holman, D. M. , Boehm, J. E. , Peipins, L. A. , Grossman, M. , & Henley, S. J. (2014). Age and cancer risk: A potentially modifiable relationship. American Journal of Preventive Medicine, 46(3 Suppl 1), S7–S15. 10.1016/j.amepre.2013.10.029 24512933 PMC4544764

[acel14174-bib-0102] Willeit, P. , Willeit, J. , Mayr, A. , Weger, S. , Oberhollenzer, F. , Brandstatter, A. , Kronenberg, F. , & Kiechl, S. (2010). Telomere length and risk of incident cancer and cancer mortality. JAMA, 304(1), 69–75. 10.1001/jama.2010.897 20606151

[acel14174-bib-0103] Wu, X. , Amos, C. I. , Zhu, Y. , Zhao, H. , Grossman, B. H. , Shay, J. W. , Luo, S. , Hong, W. K. , & Spitz, M. R. (2003). Telomere dysfunction: A potential cancer predisposition factor. Journal of the National Cancer Institute, 95(16), 1211–1218. 10.1093/jnci/djg011 12928346

[acel14174-bib-0104] Xu, K. , Zhang, X. , Wang, Z. , Hu, Y. , & Sinha, R. (2018). Epigenome‐wide association analysis revealed that SOCS3 methylation influences the effect of cumulative stress on obesity. Biological Psychology, 131, 63–71. 10.1016/j.biopsycho.2016.11.001 27826092 PMC5419875

[acel14174-bib-0105] Yu, M. , Hazelton, W. D. , Luebeck, G. E. , & Grady, W. M. (2020). Epigenetic aging: More than Just a clock when it comes to cancer. Cancer Research, 80(3), 367–374. 10.1158/0008-5472.CAN-19-0924 31694907 PMC7002254

[acel14174-bib-0106] Zee, R. Y. , Castonguay, A. J. , Barton, N. S. , & Buring, J. E. (2009). Mean telomere length and risk of incident colorectal carcinoma: A prospective, nested case‐control approach. Cancer Epidemiology, Biomarkers & Prevention, 18(8), 2280–2282. 10.1158/1055-9965.EPI-09-0360 PMC2774215 19661087

[acel14174-bib-0107] Zhang, C. , Chen, X. , Li, L. , Zhou, Y. , Wang, C. , & Hou, S. (2015). The association between telomere length and cancer prognosis: Evidence from a meta‐analysis. PLoS One, 10(7), e0133174. 10.1371/journal.pone.0133174 26177192 PMC4503690

[acel14174-bib-0108] Zhang, X. , Hu, Y. , Justice, A. C. , Li, B. , Wang, Z. , Zhao, H. , Krystal, J. H. , & Xu, K. (2017). DNA methylation signatures of illicit drug injection and hepatitis C are associated with HIV frailty. Nature Communications, 8(1), 2243. 10.1038/s41467-017-02326-1 PMC5740109 29269866

[acel14174-bib-0109] Zhang, X. , Justice, A. C. , Hu, Y. , Wang, Z. , Zhao, H. , Wang, G. , Johnson, E. O. , Emu, B. , Sutton, R. E. , Krystal, J. H. , & Xu, K. (2016). Epigenome‐wide differential DNA methylation between HIV‐infected and uninfected individuals. Epigenetics, 11(10), 750–760. 10.1080/15592294.2016.1221569 27672717 PMC5094631

[acel14174-bib-0110] Zheng, Y. , Joyce, B. T. , Colicino, E. , Liu, L. , Zhang, W. , Dai, Q. , Shrubsole, M. J. , Kibbe, W. A. , Gao, T. , Zhang, Z. , Jafari, N. , Vokonas, P. , Schwartz, J. , Baccarelli, A. A. , & Hou, L. (2016). Blood epigenetic age may predict cancer incidence and mortality. eBioMedicine, 5, 68–73. 10.1016/j.ebiom.2016.02.008 27077113 PMC4816845

[acel14174-bib-0111] Zheng, Y. L. , Ambrosone, C. , Byrne, C. , Davis, W. , Nesline, M. , & McCann, S. E. (2010). Telomere length in blood cells and breast cancer risk: Investigations in two case‐control studies. Breast Cancer Research and Treatment, 120(3), 769–775. 10.1007/s10549-009-0440-z 19543829 PMC3700420

