# Supplementary material for: DNA methylation‐based telomere length is associated with HIV infection, physical frailty, cancer, and all‐cause mortality
Source: Aging Cell. 2024 Apr 17;23(7):e14174. doi: 10.1111/acel.14174 (PMC11258465; doi:10.1111/acel.14174)
Supplement: Supplementary file 1 — Appendix S1. [file ACEL-23-e14174-s001.pdf]

Supplementary Information

DNA Methylation-Based Telomere Length Is Associated with HIV Infection, Physical Frailty, Cancer, and All-Cause Mortality

Xiaoyu Liang, Bradley E. Aouizerat, Kaku So-Armah, Mardge H. Cohen, Vincent C. Marconi, Ke Xu, and Amy C. Justice

Table S1. Demographic and clinical characteristics of WIHS.

phenotype	WIHS		
	**PWH (N=272)**	**PWoH (N=209)**	**p-value**
age	44.20 ± 8.90	39.60 ± 9.70	**1.44E-07**
BMI	28.63 ± 6.98	30.91 ± 7.61	**9.15E-04**
sex (female)	100%	100%	NA
self-reported race (AA)[1]	48.16%	50.24%	6.51E-01
smoking	62.83%	76.08%	**1.96E-03**
lnPEth[2]	NAN ± NA	NAN ± NA	NA
alcohol[3]	0.53 ± 1.22	0.95 ± 1.51	**1.83E-03**
VACS Index	NAN ± NA	NaN ± NA	NA
log10VL[4]	2.09 ± 0.56	NaN ± NA	NA
ART adherence[5]	81.04%	0%	**1.09E-69**
cannabis use[6]	30.22%	98.04%	**1.83E-19**
cocaine use[6]<br>opioid use[6]	19.40%	35.61%	**7.46E-05**
stimulant use[6]	NAN ± NA	NAN ± NA	NA
CD4T	0.21 ± 0.10	0.41 ± 0.08	**8.85E-86**
CD8T	0.28 ± 0.09	0.17 ± 0.06	**2.36E-45**
Granulocyte	0.01 ± 0.04	0.01 ± 0.05	8.17E-01
NK	0.17 ± 0.10	0.14 ± 0.07	**6.34E-03**
Bcell	0.17 ± 0.08	0.14 ± 0.05	**2.06E-07**
Monocyte	0.17 ± 0.08	0.14 ± 0.07	**5.45E-07**

[1]AA: African American; [2]lnPEth: natural logarithm of phosphatidylethanol, an objective measure of alcohol consumption; [3]alcohol: AUDIT-C (first three questions of the Alcohol Use Disorders Identification Test) in VACS and NDRNKWK (number of drinks per week) in WIHS; [4]log10VL: log10 of viral load; [5]ART adherence: adherence to antiretroviral therapy; [6]The cannabis, cocaine, stimulant, and opioid use were defined as a case (>0) and a control (=0). In WIHS, instead of individual measures for opioid or cocaine use, the phenotype is based on the cumulative use of opioids or cocaine.

Welch's two-sample t-test was used to compare means between two groups; chi-square test was used to compare percentages between two groups. Significant phenotypes are shown in bold.

Table S2. Associations between DNAmAge and HIV in VACS1 and WIHS.

Cohort	Method	adjust age	beta	beta.95CI	p	Bonferroni adjusted p	MD
VACS1	TL	no	-0.23	(-0.30, -0.16)	**1.18E-09**	**2.12E-08**	-0.37
		yes	-0.25	(-0.32, -0.18)	**1.48E-12**	**2.66E-11**	
	Mono	no	2.52	(0.17, 4.87)	**3.57E-02**	6.43E-01	8.36
		yes	3.56	(1.77, 5.36)	**1.08E-04**	**1.94E-03**	
	Horvath	no	-1.05	(-3.09, 0.99)	3.12E-01	5.62E+00	-0.43
		yes	-0.05	(-1.47, 1.37)	9.44E-01	1.70E+01	
	Hannum	no	-6.79	(-8.95, -4.63)	**1.09E-09**	**1.97E-08**	-7.78
		yes	-5.73	(-7.26, -4.21)	**3.97E-13**	**7.15E-12**	
	Pheno	no	2.69	(0.32, 5.06)	**2.63E-02**	4.73E-01	4.24
		yes	3.71	(1.86, 5.56)	**9.23E-05**	**1.66E-03**	
	Grim	no	-4.08	(-6.22, -1.94)	**1.98E-04**	**3.56E-03**	-5.34
		yes	-2.90	(-4.17, -1.63)	**8.76E-06**	**1.58E-04**	
	DunedinPoAm	no	0.06	(0.04, 0.08)	**9.75E-09**	**1.76E-07**	0.06
		yes	0.06	(0.04, 0.08)	**9.07E-09**	**1.63E-07**	
	IEAA	no	0.80	(-0.56, 2.16)	2.50E-01	4.50E+00	0.32
		yes	0.78	(-0.58, 2.15)	2.61E-01	4.70E+00	
	EEAA	no	-0.38	(-1.79, 1.02)	5.93E-01	1.07E+01	1.45
		yes	-0.44	(-1.84, 0.97)	5.43E-01	9.77E+00	
WIHS	TL	no	-0.13	(-0.21, -0.05)	**2.65E-03**	**4.78E-02**	-0.38
		yes	-0.01	(-0.08, 0.05)	6.80E-01	1.22E+01	
	Mono	no	6.35	(2.97, 9.73)	**2.61E-04**	**4.70E-03**	17.41
		yes	0.65	(-1.60, 2.90)	5.73E-01	1.03E+01	
	Horvath	no	6.49	(3.96, 9.01)	**7.11E-07**	**1.28E-05**	7.5
		yes	1.79	(0.37, 3.2)	**1.36E-02**	2.44E-01	
	Hannum	no	3.89	(1.54, 6.24)	**1.25E-03**	**2.25E-02**	6.09
		yes	-0.37	(-1.76, 1.02)	6.02E-01	1.08E+01	
	Pheno	no	7.33	(4.19, 10.47)	**6.22E-06**	**1.12E-04**	10.73
		yes	1.79	(-0.16, 3.74)	7.31E-02	1.32E+00	
	Grim	no	5.13	(2.61, 7.65)	**7.95E-05**	**1.43E-03**	4.89
		yes	0.07	(-1.04, 1.18)	9.04E-01	1.63E+01	
	DunedinPoAm	no	0.02	(0.00, 0.05)	6.75E-02	1.22E+00	0.02
		yes	0.02	(0.00, 0.05)	7.99E-02	1.44E+00	
	IEAA	no	1.70	(0.34, 3.07)	**1.47E-02**	2.64E-01	0.8
		yes	1.90	(0.51, 3.29)	**7.84E-03**	1.41E-01	
	EEAA	no	-0.98	(-2.75, 0.79)	2.80E-01	5.04E+00	6.95
		yes	0.05	(-1.70, 1.80)	9.55E-01	1.72E+01	

The self-reported race and ethnicity, body mass index (BMI), assay sample batch, smoking status, alcohol consumption (natural logarithm of PEth in VACS and number of drinks per week in WIHS), and five cell type proportions (CD4, CD8, NK, B cell, and monocyte) were adjusted.

MD: Mean Difference of the given clock between cases and controls.

Table S3. Meta-Analysis for association between DNAmAge and HIV in VACS1 and WIHS.

Method	adjust age	$beta_{VACS}$	SE_{VACS}	$beta_{WIHS}$	SE_{WIHS}	metaZ	metaP	Bonferroni adjusted p
**TL**	no	-0.23	0.04	-0.13	0.04	-6.61	**3.94E-11**	**7.09E-10**
	yes	-0.25	0.03	-0.01	0.03	-5.27	**1.38E-07**	**2.49E-06**
**Mono**	no	2.52	1.20	6.35	1.72	3.83	**1.30E-04**	**2.33E-03**
	yes	3.56	0.92	0.65	1.15	3.39	**7.02E-04**	**1.26E-02**
**Horvath**	no	-1.05	1.04	6.49	1.29	2.38	**1.74E-02**	3.13E-01
	yes	-0.05	0.72	1.79	0.72	1.71	8.74E-02	1.57E+00
**Hannum**	no	-6.79	1.10	3.89	1.20	-2.33	**1.97E-02**	3.55E-01
	yes	-5.73	0.78	-0.37	0.71	-5.33	**9.89E-08**	**1.78E-06**
**Pheno**	no	2.69	1.21	7.33	1.60	4.53	**5.86E-06**	**1.05E-04**
	yes	3.71	0.94	1.79	1.00	4.09	**4.34E-05**	**7.82E-04**
**Grim**	no	-4.08	1.09	5.13	1.29	-0.27	7.88E-01	1.42E+01
	yes	-2.90	0.65	0.07	0.57	-2.85	**4.40E-03**	7.92E-02
**DunedinPoAm**	no	0.06	0.01	0.02	0.01	5.74	**9.73E-09**	**1.75E-07**
	yes	0.06	0.01	0.02	0.01	5.74	**9.24E-09**	**1.66E-07**
**IEAA**	no	0.80	0.69	1.70	0.70	2.54	**1.10E-02**	1.98E-01
	yes	0.78	0.70	1.90	0.71	2.67	**7.52E-03**	1.35E-01
**EEAA**	no	-0.38	0.72	-0.98	0.90	-1.09	2.76E-01	4.96E+00
	yes	-0.44	0.72	0.05	0.89	-0.44	6.57E-01	1.18E+01

The self-reported race and ethnicity, body mass index (BMI), assay sample batch, smoking status, alcohol consumption (natural logarithm of PEth in VACS and number of drinks per week in WIHS), and five cell type proportions (CD4, CD8, NK, B cell, and monocyte) were adjusted.

Table S4. Associations between DNAmAge and VACS Index in VACS1.

Method	adjust age	beta	beta.95CI	p	Bonferroni adjusted p	MD
TL	no	-0.005	(-0.006, -0.004)	**5.18E-19**	**9.32E-18**	-0.19
	yes	-0.002	(-0.003, -0.001)	**2.82E-05**	**5.08E-04**	
Mono	no	0.143	(0.112, 0.174)	**6.46E-19**	**1.16E-17**	5.87
	yes	-0.006	(-0.035, 0.022)	6.67E-01	1.20E+01	
Horvath	no	0.144	(0.117, 0.171)	**6.81E-25**	**1.23E-23**	3.70
	yes	-0.005	(-0.027, 0.018)	6.95E-01	1.25E+01	
Hannum	no	0.152	(0.124, 0.180)	**1.42E-24**	**2.56E-23**	5.08
	yes	-0.002	(-0.026, 0.023)	8.88E-01	1.60E+01	
Pheno	no	0.183	(0.153, 0.214)	**5.58E-30**	**1.00E-28**	6.55
	yes	0.048	(0.019, 0.077)	**1.43E-03**	**2.58E-02**	
Grim	no	0.179	(0.152, 0.206)	**3.88E-35**	**6.98E-34**	5.18
	yes	0.007	(-0.013, 0.027)	4.87E-01	8.76E+00	
DunedinPoAm	no	0.000	(0.000, 0.001)	5.13E-02	9.23E-01	0.03
	yes	0.000	(0.000, 0.001)	**4.28E-02**	7.71E-01	
IEAA	no	-0.006	(-0.025, 0.013)	5.40E-01	9.72E+00	-0.23
	yes	-0.005	(-0.027, 0.017)	6.53E-01	1.18E+01	
EEAA	no	0.011	(-0.009, 0.030)	2.82E-01	5.08E+00	3.67
	yes	0.024	(0.002, 0.046)	**3.44E-02**	6.18E-01	

The self-reported race and ethnicity, body mass index (BMI), assay sample batch, smoking status, alcohol consumption (natural logarithm of PEth in VACS and number of drinks per week in WIHS), and five cell type proportions (CD4, CD8, NK, B cell, and monocyte) were adjusted.

MD: Mean Difference of the given clock between cases and controls.

Table S5. Associations between DNAmAge and Cancer in VACS.

Method	adjust age	beta	beta.95CI	p	Bonferroni adjusted p	MD
**TL**	no	-0.07	(-0.10, -0.03)	**1.37E-04**	**2.47E-03**	-0.07
	yes	-0.03	(-0.06, 0.00)	5.85E-02	1.05E+00	
**Mono**	no	2.7	(1.66, 3.74)	**3.64E-07**	**6.56E-06**	3.27
	yes	0.93	(0.09, 1.77)	**3.07E-02**	5.52E-01	
**Horvath**	no	2.27	(1.33, 3.21)	**2.23E-06**	**4.01E-05**	2.30
	yes	0.54	(-0.18, 1.27)	1.43E-01	2.58E+00	
**Hannum**	no	2.5	(1.51, 3.50)	**8.64E-07**	**1.55E-05**	2.27
	yes	0.76	(-0.04, 1.55)	6.21E-02	1.12E+00	
**Pheno**	no	2.32	(1.21, 3.42)	**4.35E-05**	**7.84E-04**	2.50
	yes	0.48	(-0.44, 1.40)	3.04E-01	5.48E+00	
**Grim**	no	2.83	(1.87, 3.80)	**1.08E-08**	**1.94E-07**	2.67
	yes	0.64	(0.04, 1.24)	**3.69E-02**	6.65E-01	
**DunedinPoAm**	no	0.01	(0.00, 0.02)	1.35E-01	2.44E+00	0.01
	yes	0.01	(0.00, 0.02)	2.39E-01	4.31E+00	
**IEAA**	no	0.49	(-0.20, 1.18)	1.62E-01	2.91E+00	0.49
	yes	0.51	(-0.18, 1.21)	1.47E-01	2.65E+00	
**EEAA**	no	0.63	(-0.12, 1.39)	1.01E-01	1.82E+00	0.73
	yes	0.75	(-0.02, 1.51)	5.58E-02	1.01E+00	

The self-reported race and ethnicity, body mass index (BMI), assay sample batch, smoking status, alcohol consumption (natural logarithm of PEth in VACS and number of drinks per week in WIHS), and five cell type proportions (CD4, CD8, NK, B cell, and monocyte) were adjusted.

MD: Mean Difference of the given clock between cases and controls.

Table S6. Cox proportional hazards regression analysis for assessing the association of DNAmTL with survival time.

phenotype	HR	lower.95	upper.95	p-value
**cancer+**	1.39	1.12	1.72	**2.88E-03**
**DNAmTL**	0.60	0.44	0.82	**1.42E-03**
**age**	1.04	1.03	1.06	**6.72E-10**
**self-reported race (AA)**	1.04	0.82	1.32	7.58E-01
**smoking**	1.57	1.27	1.96	**4.60E-05**
**lnPEth**	1.02	0.97	1.08	3.79E-01

DNAmTL: DNA methylation-based Telomere Length; lnPEth: natural logarithm of phosphatidylethanol, an objective measure of alcohol consumption; HR: Hazard Ratio

Table S7. Associations between DNAmAge and log10 of viral load in VACS1.

Method	adjust age	beta	beta.95CI	p	Bonferroni adjusted p
**TL**	no	-0.02	(-0.03, 0)	**4.81E-02**	8.65E-01
	yes	-0.04	(-0.05, -0.02)	**4.56E-06**	**8.21E-05**
**Mono**	no	-0.61	(-1.14, -0.08)	**2.49E-02**	4.49E-01
	yes	0.35	(-0.07, 0.77)	1.05E-01	1.90E+00
**Horvath**	no	-0.92	(-1.38, -0.46)	**1.03E-04**	**1.85E-03**
	yes	0.03	(-0.29, 0.36)	8.37E-01	1.51E+01
**Hannum**	no	-0.9	(-1.38, -0.41)	**2.91E-04**	**5.24E-03**
	yes	0.05	(-0.31, 0.41)	7.81E-01	1.41E+01
**Pheno**	no	0	(-0.54, 0.54)	9.98E-01	1.80E+01
	yes	0.96	(0.53, 1.39)	**1.42E-05**	**2.55E-04**
**Grim**	no	-1.17	(-1.66, -0.69)	**2.11E-06**	**3.80E-05**
	yes	-0.09	(-0.38, 0.21)	5.73E-01	1.03E+01
**DunedinPoAm**	no	0	(0, 0.01)	**3.37E-02**	6.06E-01
	yes	0.01	(0, 0.01)	**1.71E-02**	3.08E-01
**IEAA**	no	0.01	(-0.3, 0.33)	9.26E-01	1.67E+01
	yes	0.01	(-0.31, 0.33)	9.50E-01	1.71E+01
**EEAA**	no	0.62	(0.3, 0.93)	**1.35E-04**	**2.43E-03**
	yes	0.58	(0.26, 0.9)	**4.18E-04**	**7.53E-03**

The self-reported race and ethnicity, body mass index (BMI), assay sample batch, smoking status, alcohol consumption (natural logarithm of PEth in VACS and number of drinks per week in WIHS), and five cell type proportions (CD4, CD8, NK, B cell, and monocyte) were adjusted for in the model.

Table S8. Associations between DNAmTL and the phenotypes of interest with adjusting viral load.

phenotype	cohort	adjust age	beta	beta.95CI	p
**VACS Index**	VACS1	no	-0.01	(-0.006, -0.004)	**1.77E-18**
		yes	0.00	(-0.003, 0.000)	8.06E-02
**cancer**	VACS1	no	-0.06	(-0.10, -0.01)	**1.01E-02**
		yes	-0.03	(-0.07, 0.01)	1.62E-01

The log10 of viral load, self-reported race and ethnicity, body mass index (BMI), assay sample batch, smoking status, alcohol consumption (natural logarithm of PEth in VACS and number of drinks per week in WIHS), and five cell type proportions (CD4, CD8, NK, B cell, and monocyte) were adjusted.

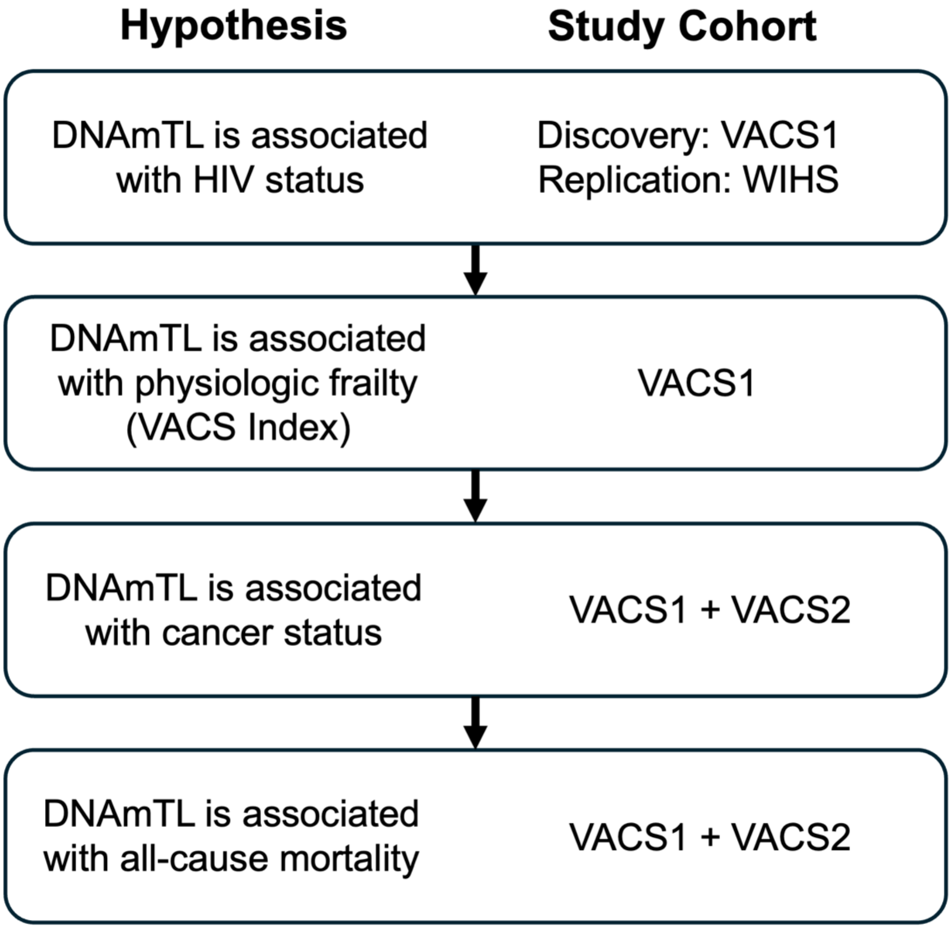

Figure S1. Hypotheses and corresponding cohorts to test the association between DNAmTL and phenotypes of interest, including HIV status, VACS Index, cancer, and mortality. VACS: Veteran Aging Cohort Study; WIHS: Women's Interagency HIV Study.

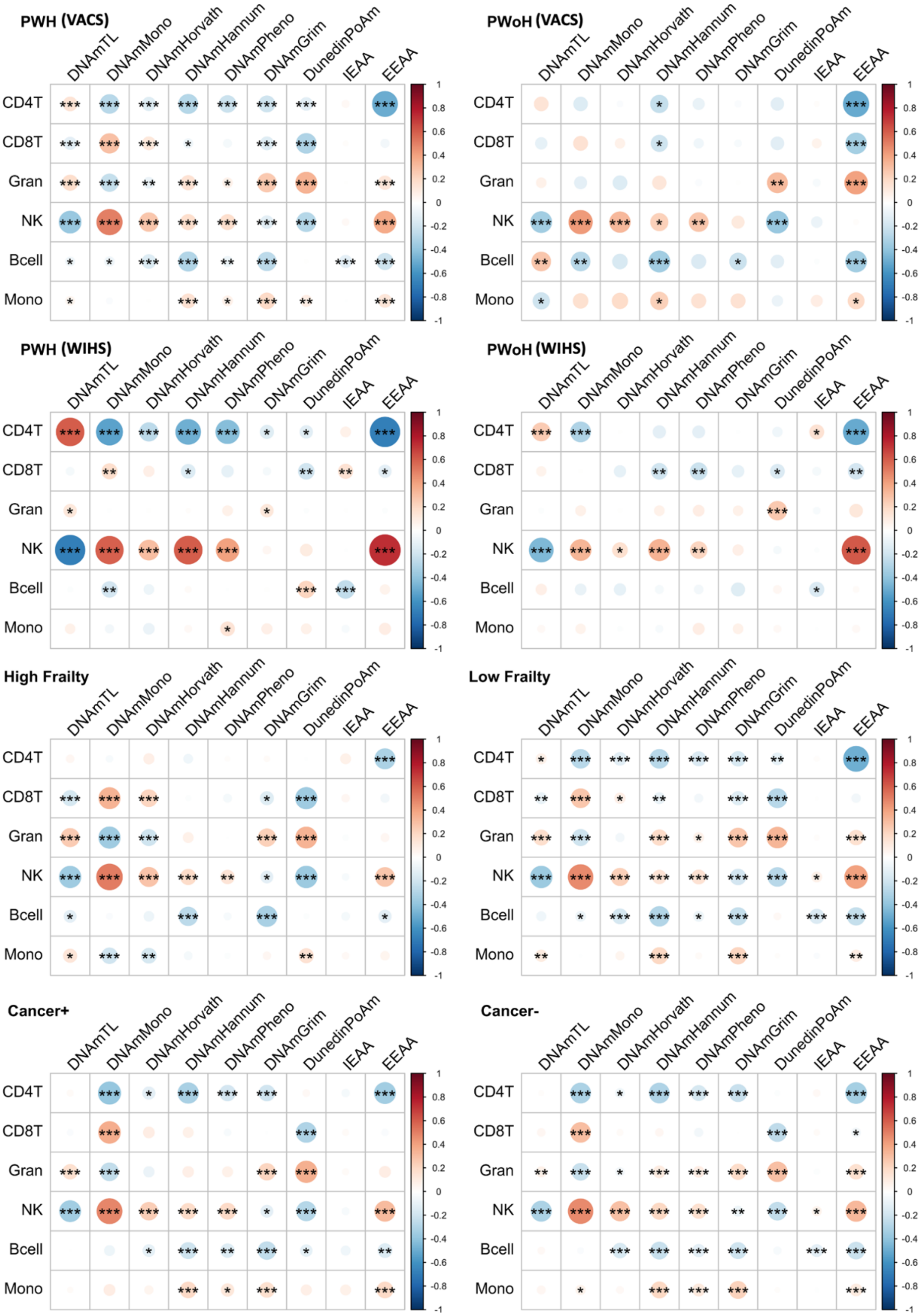

Figure S2. Correlation between epigenetic clocks and cell-type proportions. ***indicates p-value<1.00E-03; ** indicates p-value<1.00E-02; * indicates p-value<5.00E-02.

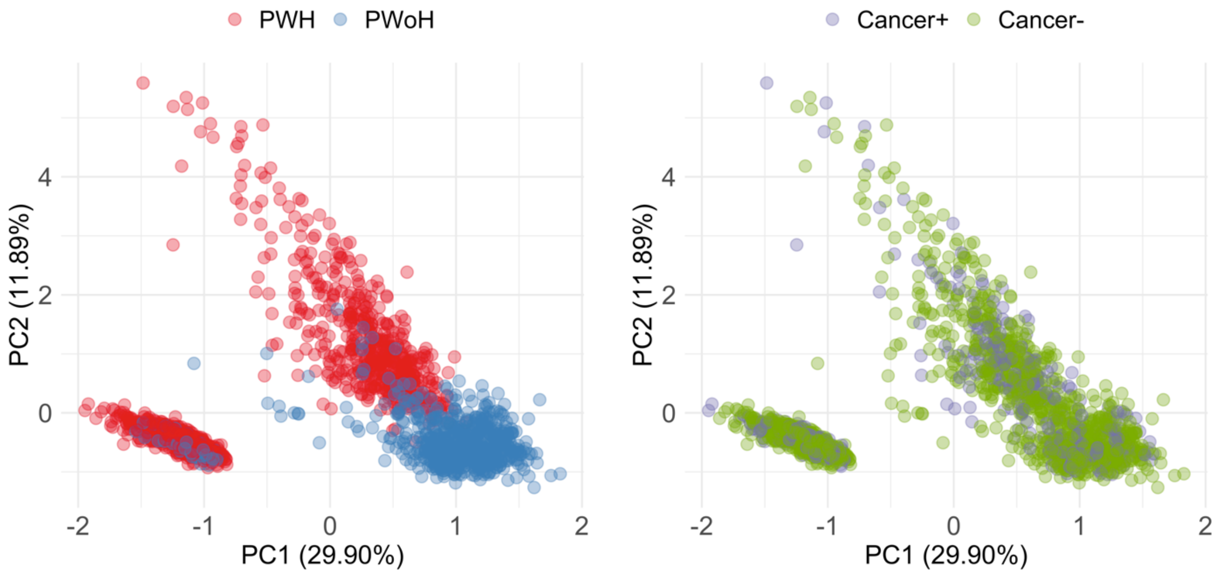

Figure S3. The Principal Component Analysis (PCA) plot displays individual samples in a reduced-dimensional space.

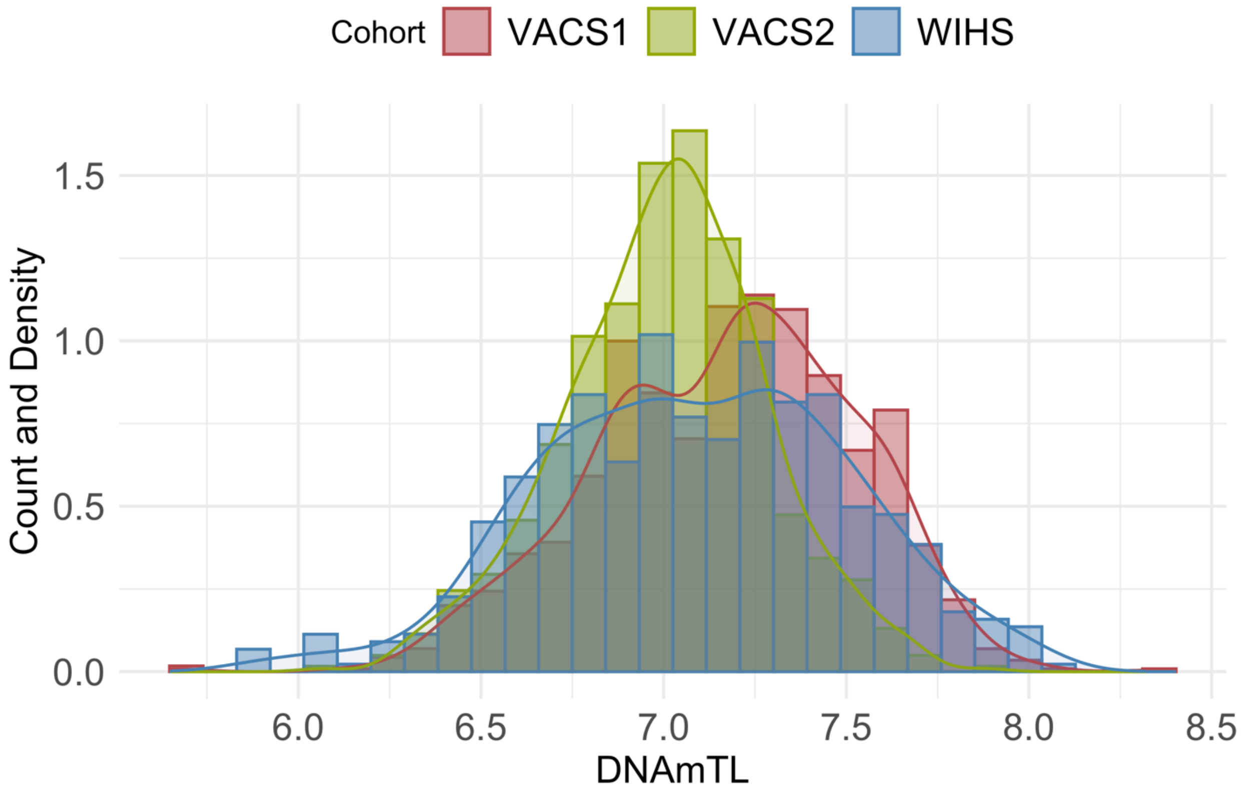

Figure S4. DNAmTL Distribution of different cohorts. VACS1: Veterans Aging Cohort Study Cohort 1, VACS2: Veterans Aging Cohort Study Cohort 2, and WIHS: Women's Interagency HIV Study.
